# 150,000-year palaeoclimate record from northern Ethiopia supports early, multiple dispersals of modern humans from Africa

**DOI:** 10.1038/s41598-018-19601-w

**Published:** 2018-01-18

**Authors:** Henry F. Lamb, C. Richard Bates, Charlotte L. Bryant, Sarah J. Davies, Dei G. Huws, Michael H. Marshall, Helen M. Roberts, Harry Toland

**Affiliations:** 1 Department of Geography and Earth Sciences, Aberystwyth University, Aberystwyth, SY23 3DB UK; 2 Department of Earth and Environmental Sciences, Irvine Building, University of St Andrews, St Andrews, Fife KY16 9AL UK; 3 NERC Radiocarbon Facility, Scottish Enterprise Technology Park, Rankine Avenue, East Kilbride, G75 0QF UK; 4 School of Ocean Sciences, Bangor University, Menai Bridge, Anglesey LL59 5AB UK; 5 Present Address: West Park School, West Road, Spondon, Derby DE21 7BT UK

## Abstract

Climatic change is widely acknowledged to have played a role in the dispersal of modern humans out of Africa, but the timing is contentious. Genetic evidence links dispersal to climatic change ~60,000 years ago, despite increasing evidence for earlier modern human presence in Asia. We report a deep seismic and near-continuous core record of the last 150,000 years from Lake Tana, Ethiopia, close to early modern human fossil sites and to postulated dispersal routes. The record shows varied climate towards the end of the penultimate glacial, followed by an abrupt change to relatively stable moist climate during the last interglacial. These conditions could have favoured selection for behavioural versatility, population growth and range expansion, supporting models of early, multiple dispersals of modern humans from Africa.

## Introduction

**Figure 1:**
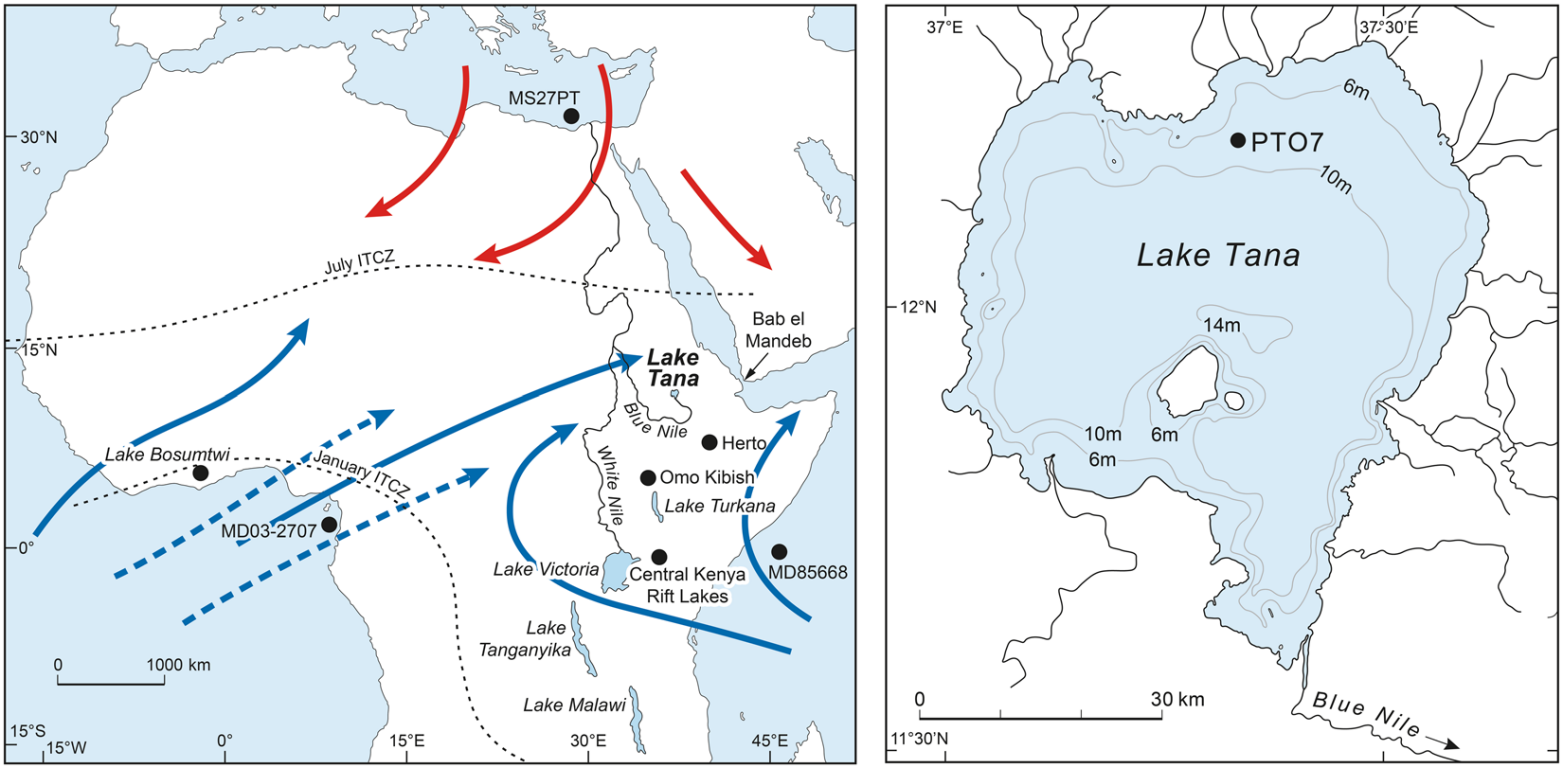
(**a**) Location and climatic context of Lake Tana. Blue arrows indicate paths of rain-bearing monsoon air masses in boreal summer, when the Intertropical Convergence Zone (ICTZ) moves northward. From October to May, the ITCZ shifts southwards and dry airflows dominate (red arrows). Marine and lacustrine core sites, earliest *Homo sapiens* sites (Herto, Omo), and Bab el Mandeb strait are also shown. (**b**) Position of core PT07 in Lake Tana at 12°N, 37° 15′ E. Maps generated in Adobe Illustrator CS6 Version:16.0.4. https://www.adobe.com/uk/.

Understanding the role of climatic change in the emergence of *Homo sapiens* (anatomically modern humans, AMH) in eastern Africa and their subsequent expansion into Asia requires continuous, well-dated terrestrial records for the relevant time range, currently lacking from the region. Palaeontological^1–3^ and genetic^4,5^ data indicate that AMH emerged around 200–300,000 years ago (ka), but there is much debate about the timing of their dispersal out of Africa^6,7^. Early dispersals during Marine Isotope Stage 5 (MIS 5; 130–90 ka) may be inferred from 90–120 kyr fossils in the Levant^8,9^, 80–120 kyr human teeth from Fuyan Cave, China^10^, 73–63 kyr teeth from Sumatra^11^, a 63 kyr cranium from Tam Pa Ling, Laos^12^, from neurocranial shape diversity^13^, and from stone artefacts at Jebel Faya, U.A.E., dated to 95–127 ka^14^. However, most phylogenetic estimates from living Eurasian populations point to their origin from a single late exodus at around 80–40 ka^15,16^ initiating AMH expansion across south Asia to Australia by ~65 ka^17^, although the data do not preclude earlier migrations whose lineages are now extinct^18^. Late AMH dispersal beyond Africa is sometimes linked to wetter, more stable climate at ~70 ka following episodes of severe aridity, apparent in the records from lakes Malawi, Tanganyika and Bosumtwi^19^. Those lakes are distant from and in different climatic regimes to the AMH fossil localities. In contrast, Lake Tana, northern Ethiopia, is close to the earliest eastern African AMH sites Herto^1^ and Omo Kibish^2^, and to postulated dispersal routes (Nile corridor, Saharan and Arabian watercourses^20,21^, Red Sea coasts and Bab el Mandeb strait^14,22^; Fig. 1a).

Summer monsoon rains, derived from the Indian Ocean and from the Atlantic via the Congo Basin^23^, feed four perennial and numerous ephemeral rivers entering Lake Tana. At 1785 m on the Tertiary flood basalts of the Ethiopian plateau, the lake occupies a 60–80 km diameter steep-sided basin, interpreted as an Oligocene caldera^24^. At its only outflow, the Blue Nile crosses scoraceous basalt with an ^40^Ar/^39^Ar age of 33 ± 5 ka^24^. Runoff from these highlands contributes most of the lower Nile discharge and a vastly greater proportion of its transported sediment^25^. Desiccation of Lake Tana at the time of Heinrich Stadial 1 (H1, ~16 ka)^26,27^ coincided with drying of Lake Victoria, source of the White Nile^28^, underlining the sensitivity of the entire Nile basin to climate extremes.

## Results

### Seismic and geochemical data

**Figure 2:**
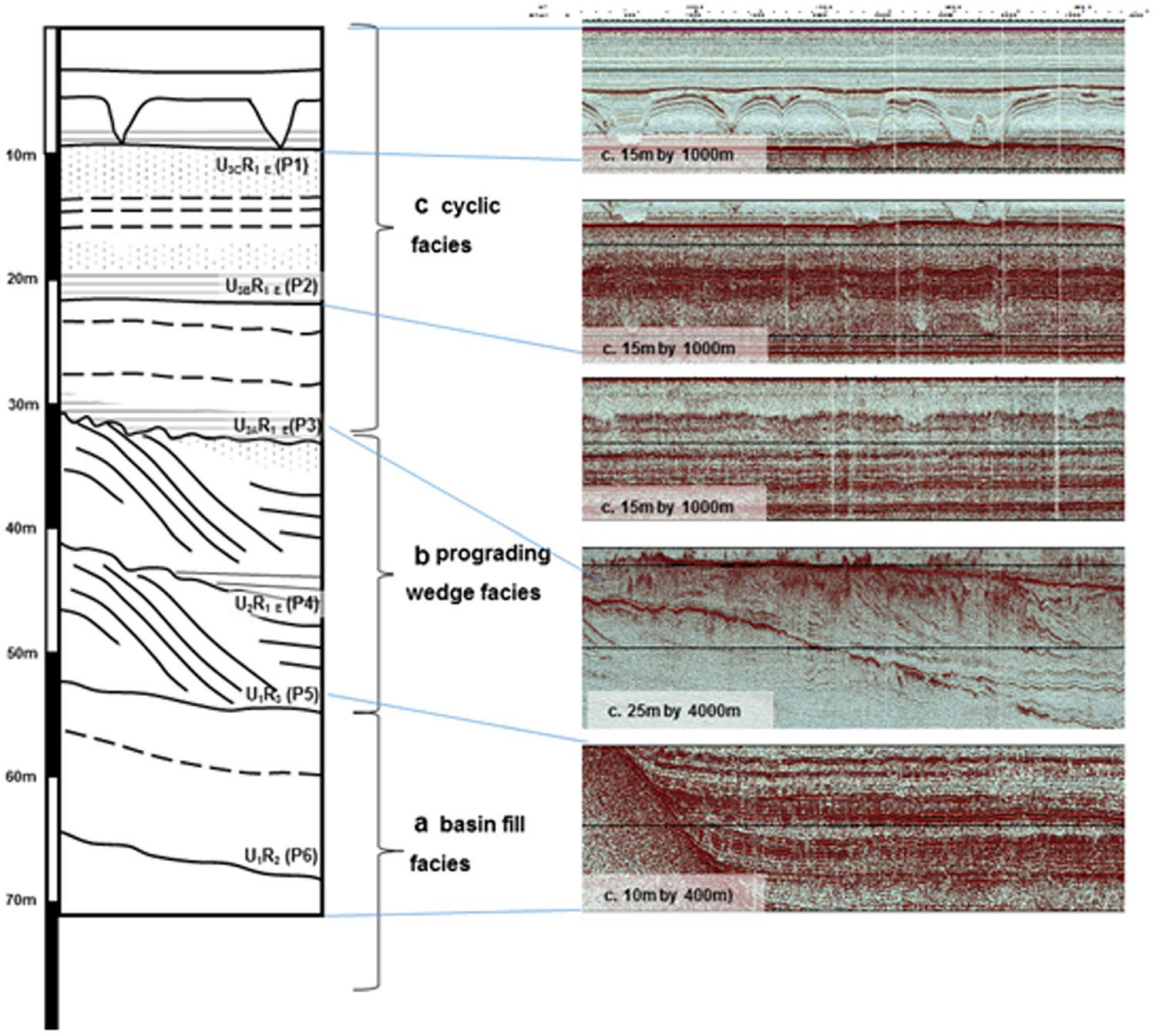
Type-section data extracts of the three major seismic facies in the vicinity of core PT07. (**a**) Basin fill facies of unit 1 showing conformable strata onlapping onto seismic basement. (**b**) Wedge facies of unit 2, showing progradational phase. (**c**) Cyclic facies of unit 3 showing repeating reflectors, and pronounced cut-and-fill features. Reflector labels as in Supplementary Table 3. Only the uppermost 70 m of the >100 m seismic profile is shown here. Seismic acquired using SonatWiz, v5, Chesapeake Inc. (https://chesapeaketech.com/) and processed with Kingdom, IHS Markit https://kingdom.ihs.com/.

**Figure 3:**
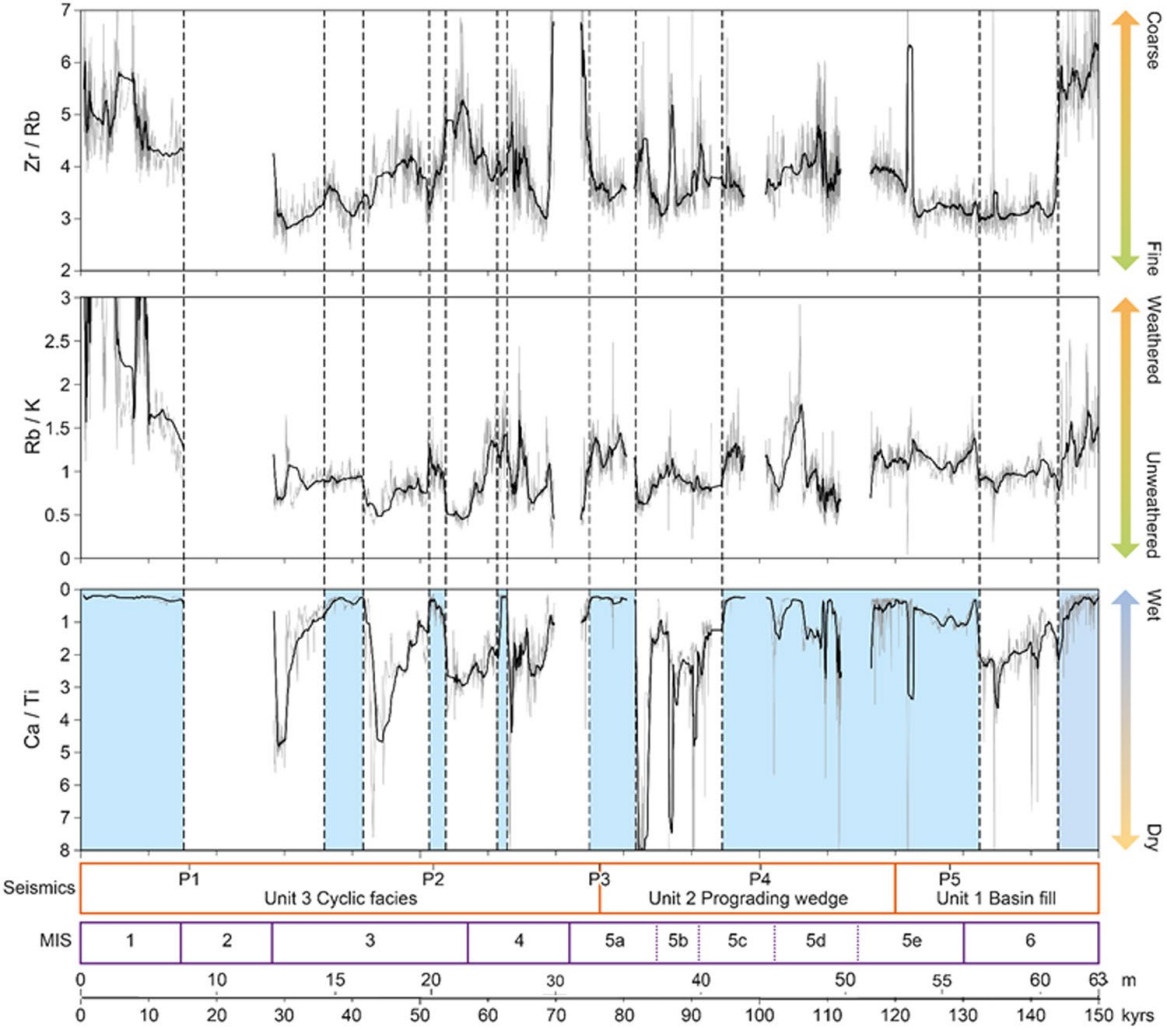
Selected elemental ratios determined by Itrax® XRF scanning of core PT07 at 2 mm intervals, plotted as 25-point running means against a 150 kyr timescale and a non-linear depth scale, with approximate boundaries of the major seismic units and Marine Isotope Stages (MIS). Blue shading indicates intervals of predominantly high lake level. Note reversed scale for Ca/Ti ratios. Figure generated in Adobe Illustrator CS6 Version:16.0.4. https://www.adobe.com/uk/.

Seismic data from Lake Tana reveal >100 m of unconsolidated sediments punctuated by strong continuous reflectors, with no apparent faulting (Fig. 2; Supplementary Methods, Supplementary Fig. 1, 13–17; Supplementary Table 3). Three seismic units contain desiccation surfaces, truncated deltaic structures, and cut-and-fill features interpreted as channels, indicating a complex sedimentation history resulting from lake-level fluctuations. The deepest seismic unit 1 onlaps the acoustic basement, interpreted as basalt bedrock, and shows a sequence of basin fill with horizontal planar reflectors, succeeded by unit 2 in which the basin fill is disrupted by unconformities and prograding wedges. The uppermost seismic unit 3 shows a repeated sequence of fill with downcutting events similar to those reported previously in the section from 18 ka to present^25,26^. Core PT07-2 reached a depth of 91.8 m. Fifteen post-IR IRSL and four radiocarbon ages indicate that the core extends to 250 ka (Supplementary Methods). Overall core recovery was 80% with most of the missing sediments from below 63 mbss. We focus here on the uppermost 62.7 m, representing the last ~150 ka. The elemental ratios Zr/Rb, Rb/K and Ca/Ti (Fig. 3) may be interpreted as proxies for sediment grain size, degree of chemical weathering, and effective moisture (precipitation minus evaporation) respectively^29^. Ti is an unambiguous indicator of allochthonous inputs, so higher values of Ca/Ti identify within-lake Ca precipitation during evaporative concentration of the lake water, lower lake levels, and thus drier climate. The catchment erosion proxies Zr/Rb and Rb/K show elevated values since ~5 ka, probably as a result of anthropogenic disturbance. In contrast, low Holocene values of Ca/Ti indicate lake overflow conditions, making a useful baseline for interpreting the older record. Short-term variations in the data reflect the hydrological sensitivity of a shallow lake, and the proximity of the core site to the lake shore (Fig. 1b).

Within age uncertainties, the seismic and Ca/Ti data are mutually reinforcing, supporting an interpretation of climate-driven lake-level change (Fig. 3). The basin fill facies (seismic unit 1, ≥55 m, ≥120 ka), interpreted as deep-water sedimentation, corresponds to initially low Ca/Ti values (high lake-level) with relatively high input of coarse and weathered sediment. At ~144 ka grain size and weathering diminish abruptly and Ca/Ti increases, indicating a drying trend with reduced erosive input. Moisture increases abruptly at 132 ka, marking the onset of generally stable high lake-level conditions during MIS 5e to 5c (~132 ka to ~95 ka), punctuated by brief dry episodes. The corresponding seismic data show conformable infill, prograding wedge phases and unconformities. In combination, the data imply increased amount and intensity of rainfall during MIS 5e–5c, causing both high lake levels and increased sediment transport to the lake.

From ~95 ka until ~28 ka (MIS 5b to MIS 2) five asymmetric intervals of increasing aridity, typically lasting 8–12 kyrs, are each followed by an abrupt change to high lake levels, which last 2–5 kyrs. The oldest and longest of these dry-wet cycles, from ~95 to ~76 ka, shows the most extreme Ca/Ti variation, recording the driest episodes of the entire record. It corresponds to the most prominent prograding wedge complex in the seismic sequence (top of unit 2b), which lies above an erosive surface (reflector P3; Supplementary Table 3), indicating that low lake level was accompanied by erosion of the underlying sediments. Subsequently, sediment transport to the lake was enhanced by aridity and reduced catchment vegetation. This cycle concludes with wet, stable conditions of approximately 5 kyrs duration, the longest such interval in the cycle set. The subsequent asymmetric Ca/Ti cycles correlate to patterns of cyclicity in the seismic facies.

### Regional comparisons

**Figure 4:**
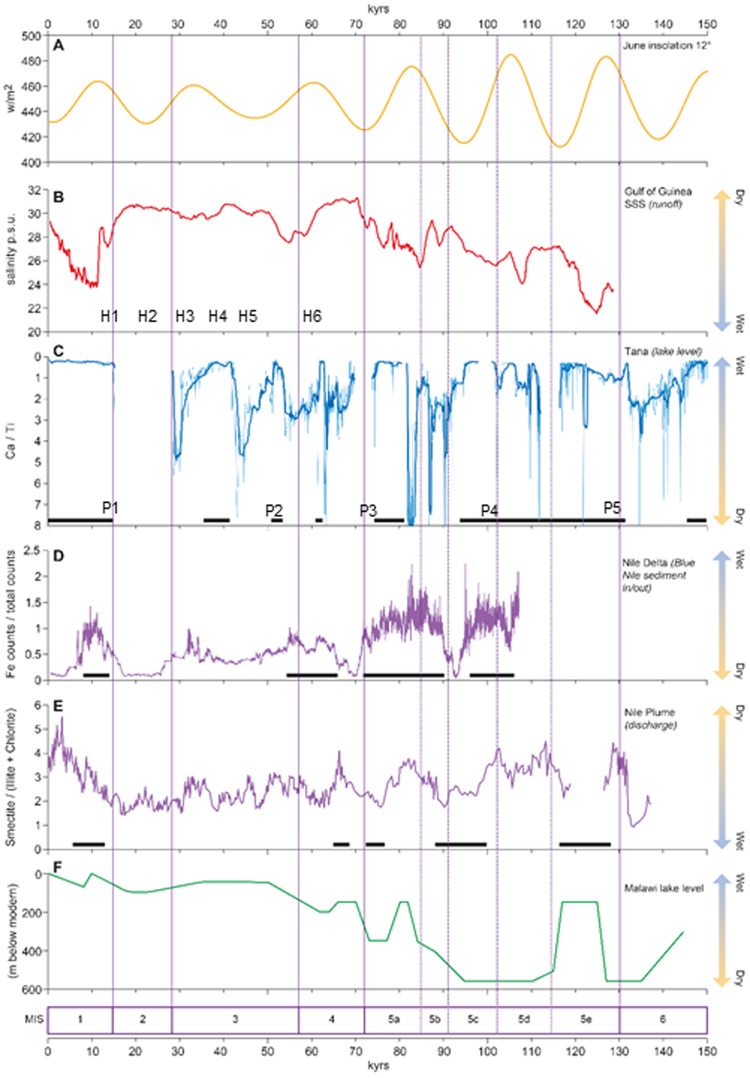
Regional expression of the climate changes at Lake Tana. (**A**) June insolation at 12° N. (**B**) Sea-surface salinity in the Gulf of Guinea, core MD03-2707^28^. (**C**) Ca/ Ti ratios, Lake Tana. (**D**) Fe XRF counts in core MS27PT, Nile delta margin^29^. (**E**) Smectite/(chlorite + illite) ratios in core Geo Tü SL110, Nile plume, eastern Mediterranean^30^. (**F**) Lake Malawi^18^. Solid bars indicate high lake levels (**C**), and high Nile discharge (**D,E**). Approximate positions of Heinrich events (H1–H6) and P-reflectors (P1–P5) shown. Figure generated in Adobe Illustrator CS6 Version:16.0.4. https://www.adobe.com/uk/.

Regional expression of the climatic changes recorded at Lake Tana is evident from similar patterns of change in west Africa and in Nile discharge records from the delta and east Mediterranean Sea, where precession-driven insolation ultimately controls millennial-scale rainfall variation and Nile discharge respectively^30–32^ (Fig. 4). These comparisons are subject to chronological uncertainties and lack of independent dating in most other records. June insolation at 12°N shows maximum amplitude during the wettest interval (132–95 ka; MIS 5e–5c) at Lake Tana, fitting models in which strong insolation increases the temperature-pressure gradient between oceans and continent, thus intensifying the north African summer monsoon. Strong fluvial runoff into the Gulf of Guinea, reflected in lowered sea-surface salinity, is also apparent during this interval^30^. Stable hydrogen isotope composition of leaf waxes in a core from the Gulf of Aden also show moist conditions in northeast Africa during MIS 5^33^. Rising values of Ca/Ti at ~82 ka indicate drought in the Blue Nile headwaters; runoff into the Gulf of Guinea was low at about the same time^30^.

The 100 kyr record of river discharge at the Nile delta^30^ shows similarities with effective moisture variation at Lake Tana, in the Nile’s principal headwaters. Nile discharge at the delta was high from >100 to 70 ka, interrupted by a dry episode at ~92 to 88 ka. Wet conditions at Lake Tana are apparent over a similar interval (~132–70 ka), but Tana’s dry episode appears to have been longer (~94–82 ka). After 70 ka, Nile discharge was generally lower than before, with two muted ‘pluvials’^29^ at ~60–50 ka and 38-30 ka. Tana also shows mostly low lake levels over this period; overspill from the lake occurred at ~54 ka and at ~42 ka, and the seismic data at 42–31 ka indicate higher lake levels; these may be linked to the delta’s ‘pluvials’. Core Geo Tü SL110 from the Nile plume in the eastern Mediterranean^32^ also shows enhanced Nile discharge during MIS 5; the abrupt increase at 132 kyr coincides with the sharp rise in the level of Lake Tana. The erosion events in Lake Tana (indicated by reflectors P4 and P3; Supplementary Table 3) occur at the end of humid intervals defined in the Nile plume record. However, Heinrich events H6–H2, marked by low Nile discharge, are not clearly apparent in the Tana record; their dates fall in a part of the record where there are many reflectors.

Glacial-age instability at Tana may be explained by diminishing eccentricity and lower precessional amplitude, with high-latitude influences increasing as ice volumes expanded. The variability of high-latitude glacial climate was apparently propagated to Tana, as shown by the frequent lake-level changes in this interval. The mechanism for this tele-connection probably involved a weaker thermohaline circulation during high-latitude stadials leading to reduced heat transport from the tropics and consequent warming of the low-latitude Atlantic, causing drought via diminished monsoon strength as the ocean-continent temperature-pressure gradient diminished. While there is a general correspondence between dry intervals in the Tana record, and ‘cold’ marine isotope stages (Fig. 4), there is less concordance with North Atlantic Heinrich and Greenland Stadials.

Where Lake Tana shows a relatively stable moist climate during MIS 5e–5c, the corresponding period at Lake Malawi (135–90 ka) is marked by two intervals when lake level fell by ~500 m, coinciding with insolation minima at this latitude^19^. Both lakes record climatic change at the start of MIS 5e, but in opposite directions: Malawi fell to 550 m below the modern level at ~135 ka, whereas Tana rose to overflow at ~132 ka. Similarly, the lakes behaved in opposite directions in MIS 5b: the level of Tana fell, while that of Malawi rose. A revised chronology for Malawi lake-level history also shows low lake level for this interval, but with less detail^34^. The contrasting but approximately coincident behaviour of the lakes can be attributed to the opposing pattern of precessional forcing in the northern and southern hemispheres: peak insolation values at 12°N coincide with minima at 12°S, their amplitude enhanced by high orbital eccentricity during MIS 5. Both records change character from late MIS 5, but again in opposite directions: Malawi shifts to wetter, more stable conditions from ~70 ka, whereas Tana shows unstable, predominantly arid conditions since ~95 ka.

### Implications for modern human dispersal

The shift to wetter conditions at ~70 ka in lakes Malawi, Tanganyika (~97.3 ka) and Bosumtwi (77.9 ka)^19^ has been linked to the expansion of early modern humans within and out of Africa. Some authors^35^ attribute population expansion and major behavioural advance within Africa between 80 and 60 ka, with subsequent dispersal to Asia, to selective pressures from varying climate at the MIS 5 - MIS 4 transition, regarding the earlier presence of modern humans in the Levant as short-lived. However, molecular data provide a wide range of dates, ~144 ka to 30 ka, for human expansion and dispersal^36^.

One model of human evolutionary response to climate variability^37^ indicates that a period of stable climate following an abrupt transition from relatively unstable conditions would favour the expansion of refugial AMH populations with the capacity to exploit marginal environments through behavioural versatility. Using similar quantitative criteria (see Supplementary Methods) we identified four stable wet intervals from the Ca/ Ti record of Lake Tana (Supplementary Fig. 18): >150–144 ka (MIS 6), 125–93 ka (MIS 5e–5c), 82-73 ka (MIS 5a), and 42–34ka (MIS 3) closely resembling simulated human dispersal intervals^38^. The longest of these, MIS 5e–5c, was preceded by an abrupt transition from variable climate in late MIS 6 that could have provided the selective pressure for versatility, and then presented long-lasting, relatively stable, moist, resource-rich conditions conducive to the demographic increase and range expansion of innovative populations into a wide range of habitats, including the Levant and Arabia^21^. The long (32 ka) duration of this interval could have allowed multiple dispersal events^39^. A similar abrupt transition at ~82 ka, from the most severe arid episode recorded to a shorter interval of stable wet conditions in MIS 5a may again have promoted population expansion and dispersal. Subsequent relatively brief wet phases at ~62 ka, ~55 ka and ~40 ka may have favoured further expansions^40^.

The early, multiple dispersal model supported here dispenses with the anomalously long time gap between modern human emergence and behavioural, technological advances in southern Africa that could have driven demographic expansion within Africa at ~80–60 ka, with subsequent dispersal into Asia^35^. The contrast between favourable interglacial conditions at Tana and contemporaneous mega-droughts at Malawi supports the view that north-eastern rather than southern Africa was the principal region of AMH increase and dispersal. Lake Tana, despite its shallow depth, shows little evidence of complete and prolonged desiccation during the ensuing glacial, suggesting that human populations could have survived in highland refugia. Overall, the Tana record shows interglacial conditions favourable for AMH increase and dispersal during the time windows suggested by recent palaeontological, archaeological and genetic data, close to likely dispersal routes into SW Asia.

## Methods

Cores 07TL-1 (10-810 cm below sediment surface), PT07-1 (2289–4202 cm bss) and PT07-2 (1315–9180 cm bss) were drilled in close proximity in January 2007 at E37° 19′ 24.3″, N12°11′56.3″, 2 km SSW of the port of Gorgora on Lake Tana’s north shore. Archive sections of the cores were scanned with an Itrax® XRF core scanner at 2 mm intervals, using a Mo X-ray tube operating at 30 kV, 50 mA with 10 second exposure times (Supplementary Methods, Supplementary Fig. 4). In the absence of terrestrial macrofossils, bulk sediment was used for radiocarbon analysis (Supplementary Methods). Results are reported as % modern ^14^C and ^14^C years BP (Supplementary Methods, Supplementary Table 1). Samples were prepared for luminescence dating according to standard methods for polymineral fine-grains (i.e. 4–11 μm diameter grains) as described in^41^. A post-IR infrared stimulated luminescence (pIRIRSL)^42^ protocol was used for dating the sediments, similar to that of ^43^. The luminescence ages (ka) (Supplementary Methods, Supplementary Table 2) were determined using the pIRIR_225_ signal calculated using the dose rates (Gy/ka) and the equivalent dose (Gy) values also shown in the table. These 15 pIRIR_225_ ages, plus four calibrated radiocarbon ages (Supplementary Methods, Supplementary Table 1), were combined to give the age-depth model (Supplementary Methods, Supplementary Fig. 5) constructed using the R-software package Bchron v. 4.1.1.

Seismic survey around the core site was undertaken using an IKB- SEISTEC^TM^ boomer operated at 105 Joules, providing a vertical resolution of 5–10 cm in the uppermost 10 m of sediments, reducing to around 50 cm at maximum penetration depth (c. 170 m). Ages were assigned to reflectors by converting the seismic two-way-travel times (TWT) of the reflectors to depths, and then converting the depths to ages using the age-depth model for the core. Detail of these procedures and the seismic results are described in the Supplementary Methods.

## Electronic supplementary material

Supplementary Information

## Data Availability

The datasets generated during the current study are deposited at the UK National Geoscience Data Centre (http://www.bgs.ac.uk/services/ngdc/).
