# Author Correction: 150,000-year palaeoclimate record from northern Ethiopia supports early, multiple dispersals of modern humans from Africa

**DOI:** 10.1038/s41598-018-37595-3

**Published:** 2018-12-17

**Authors:** Henry F. Lamb, C. Richard Bates, Charlotte L. Bryant, Sarah J. Davies, Dei G. Huws, Michael H. Marshall, Helen M. Roberts, Harry Toland

**Affiliations:** 1 Department of Geography and Earth Sciences, Aberystwyth University, Aberystwyth, SY23 3DB UK; 2 Department of Earth and Environmental Sciences, Irvine Building, University of St Andrews, St Andrews, Fife KY16 9AL UK; 3 NERC Radiocarbon Facility, Scottish Enterprise Technology Park, Rankine Avenue, East Kilbride, G75 0QF UK; 4 School of Ocean Sciences, Bangor University, Menai Bridge, Anglesey, LL59 5AB UK; 5 Present Address: West Park School, West Road, Spondon, Derby DE21 7BT UK

Correction to: *Scientific Reports* 10.1038/s41598-018-19601-w, published online 18 January 2018

Harry Toland was omitted from the author list in the original version of this Article. This has been corrected in the PDF and HTML versions of the Article, and in the accompanying Supplementary Information file.

The Acknowledgements section now reads:

“This project was funded by grants from the UK Natural Environmental Research Council (NERC; grant nos NER/B/S/2002/00540 and NE/DO12996/1) and NERC Radiocarbon Facility support (NRCF010001 allocation number 1201.1006). We thank members of the Department of Earth Sciences at Addis Ababa University for scientific and logistical support, especially the late Mohammed Umer; Addis Zeleke of Addis Geosystems plc; and the Amhara Region Agricultural Research Institute. Data are archived at the UK National Geoscience Data Centre, http://www.bgs.ac.uk/services/ngdc/home.html.”

The Author Contributions section now reads:

“H.F.L., C.R.B., S.J.D. and D.H. planned the project and undertook fieldwork in Ethiopia. M.H.M. undertook fieldwork and carried out XRF scans and laboratory analyses. H.M.R. determined luminescence dates and constructed the age model; C.B. carried out radiocarbon analyses. H.T. assisted with fieldwork and in the laboratory. All authors discussed the results and interpretation. H.F.L. wrote the manuscript with input from C.R.B., S.J.D., D.H. and H.M.R.”

