# Supplementary material for: 150,000-year palaeoclimate record from northern Ethiopia supports early, multiple dispersals of modern humans from Africa
Source: Sci Rep. 2018 Jan 18;8:1077. doi: 10.1038/s41598-018-19601-w (PMC5773494; doi:10.1038/s41598-018-19601-w)
Supplement: Supplementary file 1 — Supplementary Information [file 41598_2018_19601_MOESM1_ESM.pdf]

Supplementary Information

150,000-year palaeoclimate record from northern Ethiopia supports early, multiple dispersals of modern humans from Africa

Henry F. Lamb, C. Richard Bates, Charlotte L. Bryant, Sarah J. Davies, Dei G. Huws, Michael H. Marshall, Helen M. Roberts, Harry H . Toland

Supplementary Methods

1. Site description

Lake Tana is situated at 12°N, 37°15′E, and 1785 m altitude, on the basaltic plateau of northwest Ethiopia. Previous authors interpret its origin as a structural basin at the junction of three grabens in a structural complex that was active during the formation of the mid-Tertiary flood basalt sequence [1]. The surface catchment is dominated by Quaternary basic igneous lithologies with variety of xenoliths composed of peridotite through pyroxene, and gabbro to alkali granite[2] .The lake has a large surface area (3156 km^2), but mean and maximum depths of only 9 m and 14 m respectively. It is an oligo-mesotrophic freshwater lake (chlorophyll a average 6.4 µg l^{-1}; conductivity 220 µS cm^{-1}) with weak seasonal stratification[3] and abundant suspended sediment imparting a reddish-brown color to the water.

The lake's only surface outflow is the Blue Nile, representing about 8% of Blue Nile flow[4]. Mean annual rainfall is 1410 mm, 70% of which falls in July and August, derived from moist air masses originating from the Atlantic and Indian Ocean, when the ITCZ moves to about 19° N, just to the north of Lake Tana. From October to May, the ITCZ shifts southwards and dry conditions persist in the region.

With a maximum present-day depth of only 14 m, Lake Tana can be expected to show rapid responses to even relatively minor changes in moisture balance, but cannot record wet conditions beyond overflow level. In contrast, Lake Malawi, at the equivalent southern hemisphere latitude (12°S), is much larger and deeper: its great depth (700 m) ensures that it records a wide range of moisture variation, but with a longer response time.

2. Core retrieval

Core location was chosen based on seismic survey data showing a deep sedimentary sequence (Supplementary Figure 1). Coring was accomplished using a Boart Longyear LY38 wire-line system with a triple tube 65 mm core barrel, operated by Addis Geosystems plc from a raft anchored in 10 m water depth. We used a Livingstone piston corer to retrieve the uppermost 10 m of sediment (core 07TL-1). Overall core recovery was 80% with most of the missing sediments from below 63 mbss. Here we present core data from the sediment surface to 62.7 mbss, representing the last 150 kyr, referring to the combined cores 07TL-1 and PT07-2 as core PT-07.

3. Laboratory analyses

Cores were split into archive and working halves and held in cold storage (4°C). Lithology was logged by visual inspection and composition by smear slides under low power microscopy. Magnetic susceptibility per unit volume, κ ($\times 10^{-5}$) of the cores was measured at 2 cm intervals with a Bartington Instruments MS2 meter with a type MS2C sensor. Organic and carbonate content was estimated from percentage weight loss on ignition at 550 °C and 950 °C, respectively (Supplementary Figure 2)

Data for Si, Ca, K, Ti, Fe, Sr, and Zr were deconvolved automatically from the X-ray spectra using the Q-SPEC program. Spectra were re-evaluated following scanning to further refine the background and/or elemental peak area fitting process. Peak area element profiles were normalized by the incoherent scatter (equivalent to Compton scattering) to minimize the attenuation of the X-ray signal caused by variations in organic matter and water content (Supplementary Figure 3).

4. Radiocarbon analyses

Bulk sediment samples were digested with 2M hydrochloric acid (analytical grade, 80°C, 8 hours), washed free from mineral acid with de-ionised water then digested with 1M potassium hydroxide (analytical grade, 80°C, 2 hours). The acid-alkali soluble filtrate (following filtration via pre-combusted Whatman© GF/A filters) was discarded and the insoluble residue rinsed and heated (80°C) with deionised water followed by a final acid wash with 2M hydrochloric acid (analytical grade, 80°C, 5 hours). The residue was rinsed with deionised water and freeze dried.

Lake Tana bulk sediment has a more complex matrix and lower carbon content (0.1-1.2% after pre-treatment for samples reported here) than the bituminous coal material usually used for ^{14}C background determination at the NERC Radiocarbon Facility – East Kilbride. Deep Lake Tana sediment (i.e. beyond ^{14}C detection limits) pre-treated as above, was used to calculate a sample-specific background of 0.54+/-0.09 % modern ^{14}C (n=7) and to correct sample results. This background is equivalent to a detection limit of 0.54+2σ = 0.72 % modern ^{14}C (≥39,630 ^{14}C years BP), as defined by [5] and is higher than the background determined using bituminous coal, processed along with these samples using the same pre-treatment method. This difference may indicate greater retention of adsorbed modern atmospheric CO_2 by the clay-rich sediment, compared with the coal.

Graphite targets for ^{14}C analysis by AMS were prepared by quantitative recovery of carbon in sealed quartz tubes followed by cryogenic separation of CO_2 [6]. Aliquots of CO_2 were converted to an iron/graphite mix by iron/zinc reduction [7]. A sub-sample of CO_2 was used to measure $\delta^{13}C$ using a dual-inlet mass spectrometer with a multiple ion beam collection facility (VG OPTIMA) in order to correct ^{14}C data to –25 ‰ $\delta^{13}C_{VPDB}$. The mass spectrometer was calibrated with international reference materials to a precision of $\pm$ 0.1 ‰. ^{14}C analysis was carried out at the SUERC AMS Laboratory, East Kilbride [8]. ^{14}C enrichment of a sample is measured as a percentage (or fraction) of the ^{14}C activity relative to a modern standard (oxalic acid provided by the US National Bureau of Standards), where 100% modern is defined as the value in AD 1950, in the absence of any anthropogenic influences. Overall analytical precision is quoted at 1σ. Most samples effervesced on adding the initial acid, indicating carbonate and possible dilution of contemporary carbon (including ^{14}C) with geologically old carbonate-derived carbon (no detectable ^{14}C). This would result in an apparent lowering of ^{14}C age. The variable and relatively high $\delta^{13}C$ values suggest multiple and aquatic carbon sources in the sediment.

5. Luminescence dating

Samples were taken for luminescence dating from 65 mm diameter core sections which had previously been split and examined in daylight. The outer, light-exposed 2-3 mm of these samples were removed under subdued red lighting conditions, and used to determine the environmental dose rate (Gy/ka) shown in Supplementary Table 2. The light-safe portion of each sample was then pre-treated with 5% sodium pyrophosphate ($Na_4P_2O_7$) to deflocculate and remove a significant proportion of clay particles. Sample preparation for luminescence dating followed standard methods for polymineral fine-grains (i.e. 4-11 µm diameter grains) as described in [9]; this includes treatment with hydrochloric acid followed by hydrogen peroxide, then isolation of the 4-11 µm fraction using Stokes' Law settling in sodium oxalate. Polymineral aliquots were prepared by dispensing 1 mg of the 4-11 µm material in acetone on to each 9.8 mm diameter aluminium disc.

Luminescence measurements were made using automated Risø TL/OSL readers, equipped with infrared (IR) light-emitting diodes (LEDs; 870 nm), and $^{90}Sr/^{90}Y$ beta irradiation sources. Detection was achieved using an EMI 9235QA photomultiplier tube filtered with Corning 7-59, and Schott BG39 and GG400 filter. A post-IR infrared stimulated luminescence (pIRIRSL) [10] protocol, similar to that of [11], was used for dating but because recuperation was low (< 1 Gy in absolute terms) no high-temperature stimulation was required or employed between measurement cycles. The Single Aliquot Regenerative dose (SAR) protocol used involved preheating the sample to 250 °C for 60 s prior to stimulation with 100 s IR at a temperature of 50 °C, followed immediately by a second 100 s IR-

stimulation at an elevated temperature of 225 °C; it is this latter IRSL signal (termed the $pIRIR_{225}$ signal) that is used for dating once it has been normalised to the $pIRIR_{225}$ signal response (T_x) to a small test dose measured under the same conditions as the L_x signal. The L_x and T_x signals used for determination of the equivalent dose (D_e) were derived from the initial 1.2 s of stimulation, minus a background derived from the final 20s of stimulation.

All aliquots measured went on to be used for D_e determination and subsequent age calculations (Supplementary Table 2), each aliquot having passed a series of screening criteria, including those relating to sufficient L_x and T_x signal intensity, low recuperation (<5 % natural signal), and the ability to recycle a given dose to within 10%. Dose recovery tests using the $pIRIR_{225}$ signal and measurement protocol, gave a ratio of the recovered to given (55 Gy) dose that was within 5 % of unity. Fading tests for three aliquots of each sample gave a mean value of 0.6 ± 1.1 %/decade (n = 168; s.e. = 0.1) for the $pIRIR_{225}$ signal. No fading correction was applied as this low value is comparable with fading rates measured for quartz [12, 13], a mineral widely believed not to fade.

6. Age model

The fifteen $pIRIR_{225}$ ages generated (Supplementary Table 2*)* are in chronostratigraphic order, within uncertainties. These ages, plus four radiocarbon ages on bulk lake sediment [(a) 279 cm depth - SUERC-20086; 07TL1/3/; 2898 ± 35 BP, (b) 428 cm depth - SUERC-20087; 07TL1/5/; 6821 ± 38 BP, (c) 809 cm depth - SUERC-20090; 07TL1/9/; 12980 ± 47 BP, (d) 1400 cm depth - SUERC-16457; PT07-2/4; 26634 ± 208 BP; Supplementary Table 1], were combined to give the age-depth model shown in *Supplementary Figure 4*. The age-depth model was constructed using the R-software package Bchron v. 4.1.1 (using the algorithm of [14], and following calibration of the radiocarbon ages using Intcal13 [15]. This approach takes advantage of the law of superposition, utilising this knowledge of the relationship between samples to develop the age-depth model. The two $pIRIR_{225}$ ages which fall away from the line indicated by the 95% chronology (*Supplementary Figure 4*; sample no. 134/13 at 54 m and sample no. 134/9 at 29 m; Supplementary Table 2) agree with the overlying ages within 2 sigma uncertainties and hence are not flagged as outliers, but are instead taken to indicate periods of extremely rapid sediment accumulation. Interestingly, these two $pIRIR_{225}$ ages occur at the transitions between the three major seismic units identified in the main paper.

7. Conversion of seismic travel times to ages

Assigning ages to reflectors is a two stage process: (i) convert the seismic two-way-travel times (TWT) of the reflectors, as they appear at the core site, to depths (ii) convert the depths to ages using the age-depth model noted previously. For the former, the variation of seismic velocity with TWT must be known.

Using the equation given by [16], a velocity of 1489 m/s was calculated for the water column (for a measured water temperature of 22°C and average water depth of 10m). Thus, the lake floor reflector TWT was converted to depth. For the lake sediment, the core was logged using hand-held, field-rugged transducers comparable to the laboratory versions described by [17], with measured seismic velocities being corrected for sediment temperature. Such a technique readily detects velocity variation caused by lithological change, but in making measurements on the extracted core barrels at ambient (atmospheric) pressure, the velocity may not be representative of *in situ* values at higher effective stress. However, the data did confirm that the material was well-saturated: data quality was generally good, and this is only possible if the saturation is high.

To investigate the problem of stress-relief inducing potentially lowered velocity values, a sample of the core material from 30.5 m depth was tested using an instrumented oedometer (or consolidation) cell [18] allowing velocity variation over a range of porosity and pressure to be

investigated. To perform this test, the core material was remoulded and water added so that the sample moisture content was c. 1.3 times its liquid limit [19], as recommended by [20]. The initial laboratory sample height was 30 mm. Nine increments of load were imposed, ranging from c. 1.5 kPa to 2332 kPa. Each load was applied for 24 hours, in-turn. The cessation of primary consolidation following the imposition of each increasing load was determined by monitoring the sample height (measured to ± 0.005 mm) using a linear variable differential transducer (LDVT). At the end of each load, a seismic velocity was measured by a time-of-flight technique, with travel-time measured to ± 0.05 μs using transducers operating at 500 kHz. The sample was then incrementally *unloaded* and a corresponding sequence of velocity data acquired. This approach takes the sediment through a loss of water by compression, not unlike the processes of sediment burial or desiccation experienced *in situ*, although the extent of maximal compression (2332 kPa) was limited by the physical design of the test apparatus. The effects of secondary consolidation and aging are not accounted for.

7.1 Conversion of seismic travel times to ages: results

Measurements of sample height at the end of each successive load allowed the calculation of void ratio. This is the standard measure of void space as used in geotechnical engineering and is the proportion of void space relative to space occupied by grains [19]. The variation of void ratio with pressure is plotted in Supplementary Figure 5. A decrease in void ratio is observed as successively greater loads are added and, as is typically the case, the process of unloading induces a slight rebound of the sample, shown in the data as an increase in void ratio with decreasing load.

Also plotted are the '*in situ*' void ratio data from the core itself. The latter are derived as follows. The raw moisture content values (ω) from the core are assumed to represent saturated values. The potential for under-saturation noted with respect to initial attempts at measuring the seismic velocity is assumed to be small. This being the case, then the void ratio (*e*) can be calculated as:

$$e = \omega G_s \quad (1)$$

Where *Gs* = specific gravity of the material

In order to estimate the 'in situ' pressure at any depth, the void ratio and specific gravity can be used to calculate the saturated density (ρ_{sat}) of the material:

$$\rho_{sat} = ((G_s + e) / 1 + e)\, \rho_{water} \quad (2)$$

The core can then be treated as a series of layers, each data point of moisture content representing the centre point of a layer of density, ρ_{sat}, and thickness, *z*, (related to the spacing of the moisture content values). The effective stress contributed by each such layer can be calculated from:

$$\sigma' = (\rho_{sat} - \rho_{water}) gz \quad (3)$$

Thus, an accumulating total of effective stress (pressure) derived down the core dataset can be calculated. In this way, the moisture content and depth raw data can be converted to void ratio and effective stress and shown in conjunction with the laboratory data (**Supplementary Figure 5**). The assumptions made in this approach are:

1. The samples were saturated when moisture content measurements were performed
2. The specific gravity of the grains average 2.65.

It is clear from Supplementary Figure 5 that the field data values from the low effective stress values near the core top possesses a very high void ratio and moisture content – the latter being far in excess of the liquid limit measured for the test sample. The deeper core section field data show much lower void ratio values, some variation in values and a void ratio – log pressure gradient close to the *unload* cycle of the laboratory data. This gradient implies that the material in the core has been previously loaded i.e. is *over-consolidated*. This loading can be of the form of previous burial by sediment that has subsequently been removed, or it can result from a desiccation-induced lowering of the water table.

The seismic, or p-wave velocity (*Vp*) values during this process are shown in Supplementary Figure 6. Minimum values are observed early in the loading process, but values increase notably above 100 kPa, attaining c. 1950 m/s at maximum load and remaining above 1700 m/s for the remainder of the unload cycle, save for the final 'top cap' load.

The relationship between Supplementary Figure 5 and Supplementary Figure 6 is clarified somewhat in Supplementary Figure 7, plotting void ratio versus *Vp*. At void ratio values of between 1 and 2, the data imply that the compressional wave velocity is not strongly controlled by whether or not the data are acquired on the load as opposed to the unload cycle. (The deviation between values at the higher void ratios in the range can be explained by insufficient time being allowed for fully unloaded conditions to have developed in the laboratory test sample.)

This prime observation means that void ratio alone may be used as a reasonable empirical predictor of velocity, irrespective of the consolidation state of the material. A best-fit linear relationship is fitted for the data where $1 < e < 2$:

$$V_p = 2246.6 - 294.2\, e \qquad (4)$$

However, the data down the core indicate that the void ratio values show a wider range of values than 1 to 2. It should also be noted that the above data is representative of *one* sample point. Given these caveats, a parsimonious approach is taken:

1. A linear best-fit is ascribed to the core void ratio versus depth data so as to produce a smoothed version of the data (Supplementary Figure 8)
2. Equation (4) is used to predict the change in velocity with depth, given that void ratio at each depth is known from step 1
3. From the velocity-depth information, TWT as a function of depth can then be predicted.
4. Knowing this relationship, any TWT ascribed to a seismic reflector can be converted to depth.
5. The depth can be converted to predicted age on the basis of the BChron model.

7.2 Testing the sensitivity of predicted dates for any given depth

The velocities to be applied are clearly best estimates only. For that reason, it is prudent to test the sensitivity of the predicted date of any seismic travel time to the applied velocity value. To do this, we take the following approach.

The calculations described previously that relate travel time to age are repeated, but the velocity values are changed by ± 30 m/s compared to the best estimate for velocity. The precision of the timing instrument was noted as ± 0.05 [illegible]s; an error of ± 30 m/s is equivalent of a timing error of ± 0.15 [illegible]s. The chosen velocity error is compatible with the premise of fitting a linear relationship between laboratory oedometer measured velocity and void ratio, as given in equation (1). However, the tests assume a persistent bias of +30 m/s or -30 m/s, so that errors accumulate with travel-time or depth, making the approach rather conservative.

For any given TWT, then a range of predicted depths and hence ages will result from the ± 30 m/s range in velocity. Supplementary Figure 9 shows how this range varies with travel time. These data can also be plotted against the age (Supplementary Figure 10), as calculated by converting travel time to depth using the best velocity estimate. The general increase in sensitivity to velocity down the core is due to the accumulating effect of applying a different velocity as we convert data from travel time to depth. The pinnacles of increased difference in predicted age are an artefact of the nature of the way that the BChron model, (Supplementary Figure 4), deflects in age-depth gradient in the proximity of the underlying age-depth date points.

The predicted uncertainty in age that results from an uncertainty of 30 m/s in velocity at less than 5 ka for the first 150 ka, and is generally less than 4 ka.

8. Seismic survey results

Three phases of seismic acquisition were accomplished in Lake Tana. The first acquisition [21] used a Chirp Sub-bottom profiling system. The second acquisition used the methodology described above and was mainly concentrated on areas of high quality sequence in the north part of the lake

away from the influence of gas within the sediments. Seismic acquisition lines for the different surveys are shown in Supplementary Figure 11.

Following previous investigations into the top 40 m of section [21], the seismic data have been sub-divided into three main units. The seismic basement (U_0R_0) was recognised around the lake margin into which no energy penetration was possible. This is implied to underlie all sections where there is sufficient energy for penetration to depth. Above this, three sequences of sedimentary units are identified. Unit 1, at depths generally greater than 50 m below present lake level is interpreted as basin fill. Unit 2 is characterized by prograding wedges of sediment at the basin edge and Unit 3, from depths of approximately 40 m below present lake level to surface, comprises of a series of sub-units of repeating or "cyclic" facies. Each unit is described in detail below and summarised in Supplementary Table 3.

Seismic Basement *(Supplementary Figure 12)*

The seismic basement (reflector U_0R_0) is evident along the northern coastline as a high amplitude reflector forming the upper surface of a unit of chaotic internal character (of no lower defined limit; Supplementary Figure 12). The upper bounding reflector is manifest as a highly irregular surface that is consistent in character, dip and morphology with the bedrock evident onshore and on the many islands within the lake. Therefore, although not directly sampled, it is assumed that the reflector represents buried (basaltic) bedrock. Overlying seismic reflectors terminate by onlap on to this basement (at various angles of dip).

Unit 1 *(Supplementary Figure 13)*

Unit 1 consists of a series of near horizontal sub-parallel, continuous, low to medium amplitude internal reflectors, which gently dip upwards in an onshore direction, terminating by onlap onto the seismic basement (where the latter is present in the data) (Supplementary Figure 13). Some of the internal reflectors are slightly hummocky in nature (U_1R_{1E}) and others of more continuous uniform character. Because of its internal reflector characteristics, Unit 1 is interpreted as a *basin fill* facies.

The reflector defining the precise boundary between Unit 1 and 2, labelled U_1R_4 is a locally discontinuous, medium amplitude event that dips in the same trend as the underlying reflectors.

Unit 2 *(Supplementary Figures 14, 15)*

The overlying sub-Unit 2a is also present throughout the survey area and is sub-divided into two parts. Proximal to the present-day Megech river mouth, the lower part contains internal reflector geometry that differs markedly from the underlying Unit 1, being clinoformal in nature and dipping towards the south (Supplementary Figure 14). Lower (landward) clinoforms tend to be oblique tangential, changing to oblique parallel forms in overlying events. Though the data quality diminishes offshore, the oblique parallel nature of the internal reflectors are apparent in all dip sections proximal to the Megech. The upper terminations of the clinoforms are ambiguous in the near-shore area, being either erosional truncation or toplap. Further offshore, they show no hint of toplap and are interpreted as erosional truncation. The terminations at the base of the clinoforms are similarly ambiguous, being inferred by extrapolation to be downlapping onto the basal U_1R_2 event (due to poor signal-to-noise ratio and resolution in the data, the actual termination points are not discernible). This sub-unit is interpreted as a prograding wedge formation with some localised channelling. The upper boundary of the lower sub-Unit is marked by a strong reflector, U_2R_{1E} (P4 in [13]), which occurs

throughout the area as a medium to high amplitude, continuous, near horizontal reflector. This erosion surface cuts into the sub-unit below truncating the clinoforms.

The sub-unit 2b is bound at the base by U_2R_{1E} (P4 in (*56*)) and at the top by reflector $U_{3A}R_{1E}$, another erosion event (labelled as P3 in [13]) (Supplementary Figure 15). A lakeward and downward shift in this onlap is apparent at the base of the sub-unit. The basal strata of the sub-unit comprises continuous, slightly hummocky, near parallel, medium to low amplitude internal reflectors that updip gently shoreward, onlapping onto U_2R_{1E}. The upper reflector of this sequence of reflectors is a downlap surface for internal clinoforms that form an upper set of strata that form a prograding wedge.

If the top and bottom sets of the clinoforms for both sub-units are interpreted as representing syn-depositional lake level and lake floor respectively, then a minimum lake depth of 10-15 m (before sediment compression on burial) can be estimated for each of the prograding wedge sequences.

The upper bounding reflector $U_{3A}R_{1E}$ (labelled as P3 in [13]) of the upper sub-unit is present over most of the survey area, manifest as a continuous, medium to high amplitude event, downdipping in an offshore direction. Above the upper, shoreward part of the wedge, $U_{3A}R_{1E}$ becomes chaotic in nature and is interpreted as an erosion surface.

Unit 3 (Supplementary Figures 16, 17)

Overlying reflector $U_{3A}R_{1E}$ (P3 in [13]) are three sub-units that show a marked cyclicity in seismic facies. Each sub-unit is separated by single, high amplitude, continuous, near horizontal reflector ($U_{3B}R_{1E}$ and $U_{3C}R_{1E}$). Within each sub-unit, infilled down-cutting erosional channel-like features are apparent, for example $U_{3C}R_5$.

In all sub-units, the following facies are observed, though not necessarily in a specific stratigraphic order:

i. Continuous, horizontal, parallel, medium to high amplitude reflector sets that are present consistently, above the high amplitude basal reflector
ii. Cut-and-fill 'channel' facies are present in all three units, though they are smaller in sub-unit 3a and 3b than in 3c. In sub-unit 3a and 3b the infilling sub-unit is reflection free whereas in 3c the fill consists of onlapping reflectors.
iii. Immediately below the upper boundary, the sub-units are either seismically transparent (3a and 3b) or have only relatively low amplitude internal reflectors (3c).

There are differences between the units:

i. Sub-units 3a and 3b generally display more back-scattered seismic energy (in the form of incoherent background noise) than 3c, which is interpreted as being due to an increased density in the lower two units.
ii. Sub-units 3a and 3b also have more medium to high amplitude sub-parallel reflector sets than 3c
iii. Over the core site, where seismic line spacing was 50m, it was possible to map the extrapolated 3-D morphology of the 'channels' in sub-units 3b and 3c. These features were reported on in [22]. In sub-unit 3a, mapping reveals that the features are present as coherent channels (Supplementary Figure 17a), measuring c. 75m across and c.1 m depth. However, the cut-and-fill features in sub-unit 3c do not join coherently between sections. Instead, they form isolated basin features measuring c. 80m x 150m x 2m (Supplementary Figure 17b).

9. <u>Identifying stable wet intervals from the Ca/ Ti record of core PT07</u>

To identify stable moist intervals conducive to AMH dispersal, we followed the method of [23] but with the revised age model described above, over a longer time interval (150 – 0 ka) We resampled the Ca/Ti – age data to produce data points every 25 years, then smoothed the data to a centre-weighted Gaussian 1025 yr running mean. We defined stability as the difference between the smoothed and original values; smaller numbers imply greater stability. An age point is designated as being suitable for dispersal if (i) the unsmoothed Ca/Ti is less than half the average for the whole dataset (i.e. wet); and (ii) the calculated stability number is also less than the average (stable). The resulting stable moist intervals are indicated by the red horizontal bars in Supplementary Figure 18. For all threshold values of Ca/Ti below average (increased wetness), and allowing for missing data, MIS 5 appears to be the longest time window conducive to dispersal.

Supplementary References

1. J. Chorowicz *et al.*, The Tana basin, Ethiopia: intra-plateau uplift, rifting and subsidence. *Tectonophysics* **295**, 351-367 (1998).
2. D. Ayalew *et al.*, Source, genesis, and timing of giant ignimbrite deposits associated with Ethiopian continental flood basalts. *Geochimica Et Cosmochimica Acta* **66**, 1429-1448 (2002).
3. R. B. Wood, J. F. Talling, Chemical and Algal Relationships in a Salinity Series of Ethiopian Inland Waters. *Hydrobiologia* **158**, 29-67 (1988).
4. D. Conway, A water balance model of the Upper Blue Nile in Ethiopia. *Hydrolog Sci J* **42**, 265-286 (1997).
5. M. Stuiver, H. A. Polach, Reporting of C-14 Data - Discussion. *Radiocarbon* **19**, 355-363 (1977).
6. T. W. Boutton *et al.*, Comparison of Quartz and Pyrex Tubes for Combustion of Organic-Samples for Stable Carbon Isotope Analysis. *Anal Chem* **55**, 1832-1833 (1983).
7. P. J. Slota, A. J. T. Jull, T. W. Linick, L. J. Toolin, Preparation of Small Samples for C-14 Accelerator Targets by Catalytic Reduction of Co. *Radiocarbon* **29**, 303-306 (1987).
8. S. P. H. T. Freeman, A. Dougans, L. McHargue, K. M. Wilcken, S. Xu, Performance of the new single stage accelerator mass spectrometer at the SUERC. *Nucl Instrum Meth B* **266**, 2225-2228 (2008).
9. H. M. Roberts, Testing Post-IR IRSL protocols for minimising fading in feldspars, using Alaskan loess with independent chronological control. *Radiat Meas* **47**, 716-724 (2012).
10. K. J. Thomsen, A. S. Murray, M. Jain, L. Botter-Jensen, Laboratory fading rates of various luminescence signals from feldspar-rich sediment extracts. *Radiat Meas* **43**, 1474-1486 (2008).
11. J. P. Buylaert, A. S. Murray, K. J. Thomsen, M. Jain, Testing the potential of an elevated temperature IRSL signal from K-feldspar. *Radiat Meas* **44**, 560-565 (2009).
12. C. Thiel *et al.*, Luminescence dating of the Stratzing loess profile (Austria) - Testing the potential of an elevated temperature post-IR IRSL protocol. *Quaternary International* **234**, 23-31 (2011).
13. J. P. Buylaert *et al.*, A robust feldspar luminescence dating method for Middle and Late Pleistocene sediments. *Boreas* **41**, 435-451 (2012).
14. J. Haslett, A. Parnell, A simple monotone process with application to radiocarbon-dated depth chronologies. *J R Stat Soc C-Appl* **57**, 399-418 (2008).
15. P.J. Reimer et al. IntCal13 and Marine13 radiocarbon age calibration curves 0–50,000 years cal BP. Radiocarbon 55, 1869–1887 (2013).
16. A. B. Coppens, Simple Equations for the Speed of Sound in Neptunian Waters. *J Acoust Soc Am* **69**, 862-863 (1981).
17. P. J. Schultheiss, P. P. E. Weaver, Multisensor Core Logging for Science and Industry. *Oceans 92 : Proceedings, Vol 1 and 2*, 608-613 (1992).
18. F. A. I. Hamdi, D. T. Smith, Soil Consolidation Behavior Assessed by Seismic Velocity-Measurements. *Geophys Prospect* **29**, 715-729 (1981).

19. J. Knappett, R. F. Craig, R. F. Craig, *Craig's soil mechanics*. (Spon Press, Abingdon, Oxon ; New York, ed. 8th, 2012), pp. xxv, 552 p.
20. J. B. Burland, 30th Rankine Lecture - on the Compressibility and Shear-Strength of Natural Clays. *Geotechnique* **40**, 329-378 (1990).
21. C. R. Bates, H. F. Lamb, M. Umer, High-resolution seismic investigation of Lake Tana, northern Ethiopia. *Near Surf Geophys* **5**, 243-250 (2007).
22. D. Phillips, Bates, C.R., Geophysics and climate research: High resolution 2-D seismic surveys recorded at Lake Tana, Ethiopia - The source of the Blue Nile. *SEG Technical Program Expanded Abstracts*, 1328-1331 (2008).
23. M. Grove *et al.*, Climatic variability, plasticity, and dispersal: A case study from Lake Tana, Ethiopia. *J Hum Evol* **87**, 32-47 (2015).
24. J. R. Prescott, J. T. Hutton, Cosmic-Ray Contributions to Dose-Rates for Luminescence and Esr Dating - Large Depths and Long-Term Time Variations. Radiat Meas 23, 497-500 (1994).
25. G. Adamiec, Aitken, M., Dose-rate conversion factors: update. *Ancient TL* **16**, 37-49 (1998).
26. J. Rees-Jones, Optical dating of young sediments using fine-grain quartz. Ancient TL 13, 9-14 (1995).

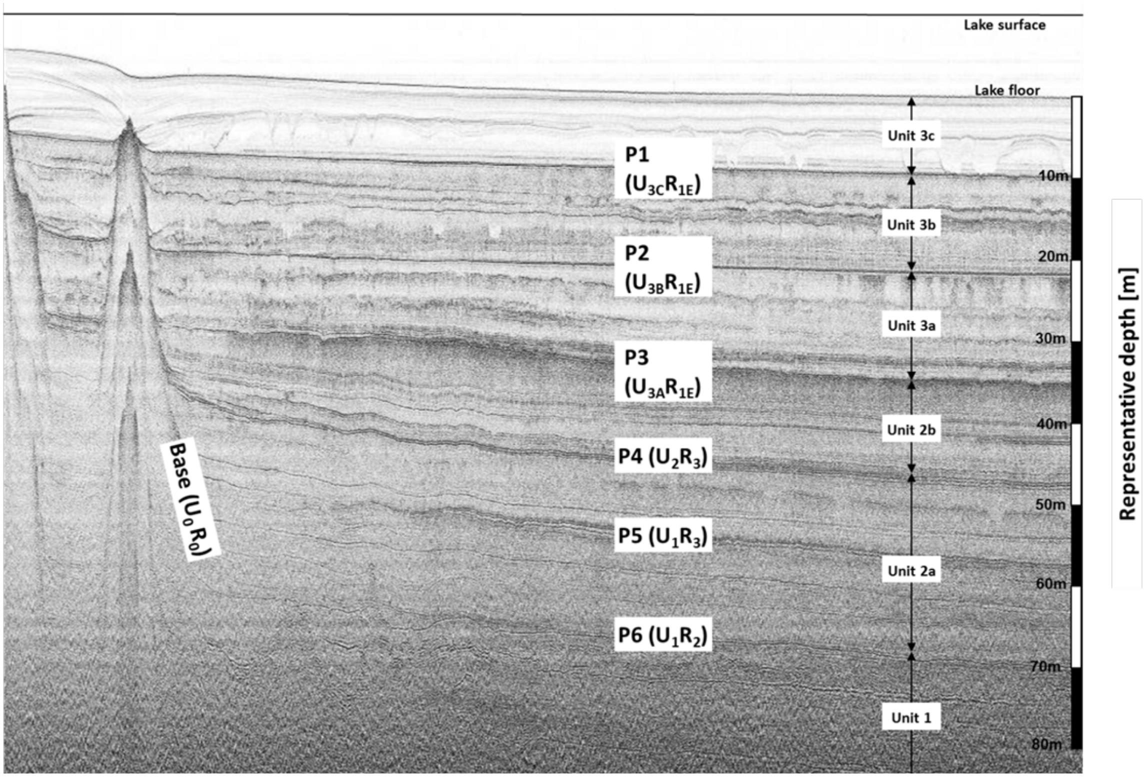

Supplementary Figure 1: Generalized seismic section for northern Lake Tana. Details of seismic reflectors and seismic units are given in Supplementary Table 3.

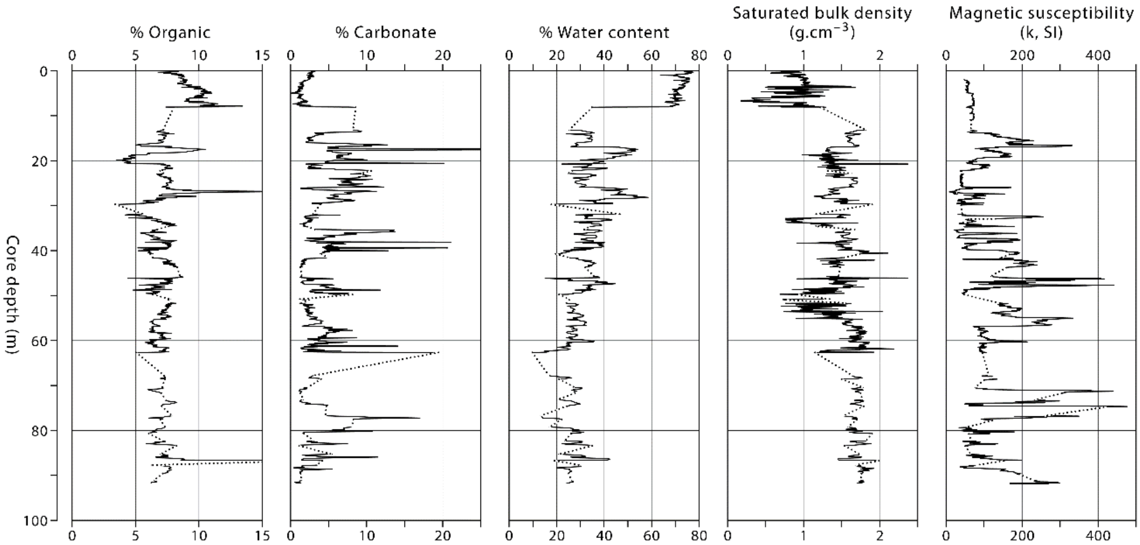

Supplementary Figure 2: Core PT07 composition and magnetic susceptibility.

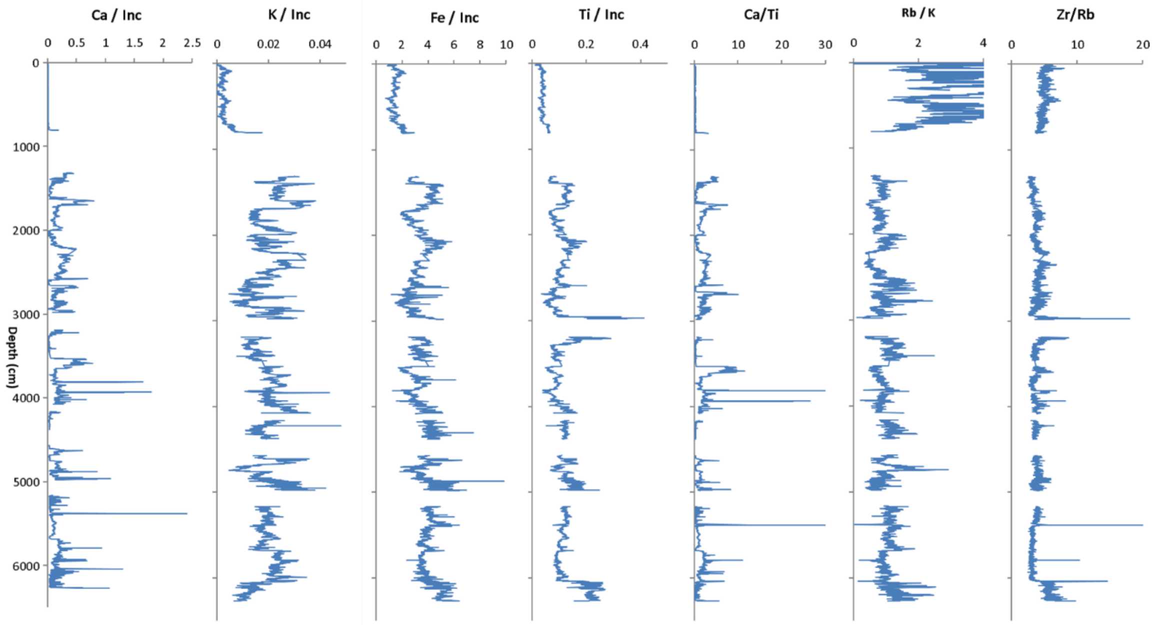

Supplementary Figure 3: Core PT07: selected XRF scan data, 0- 60 m bss.

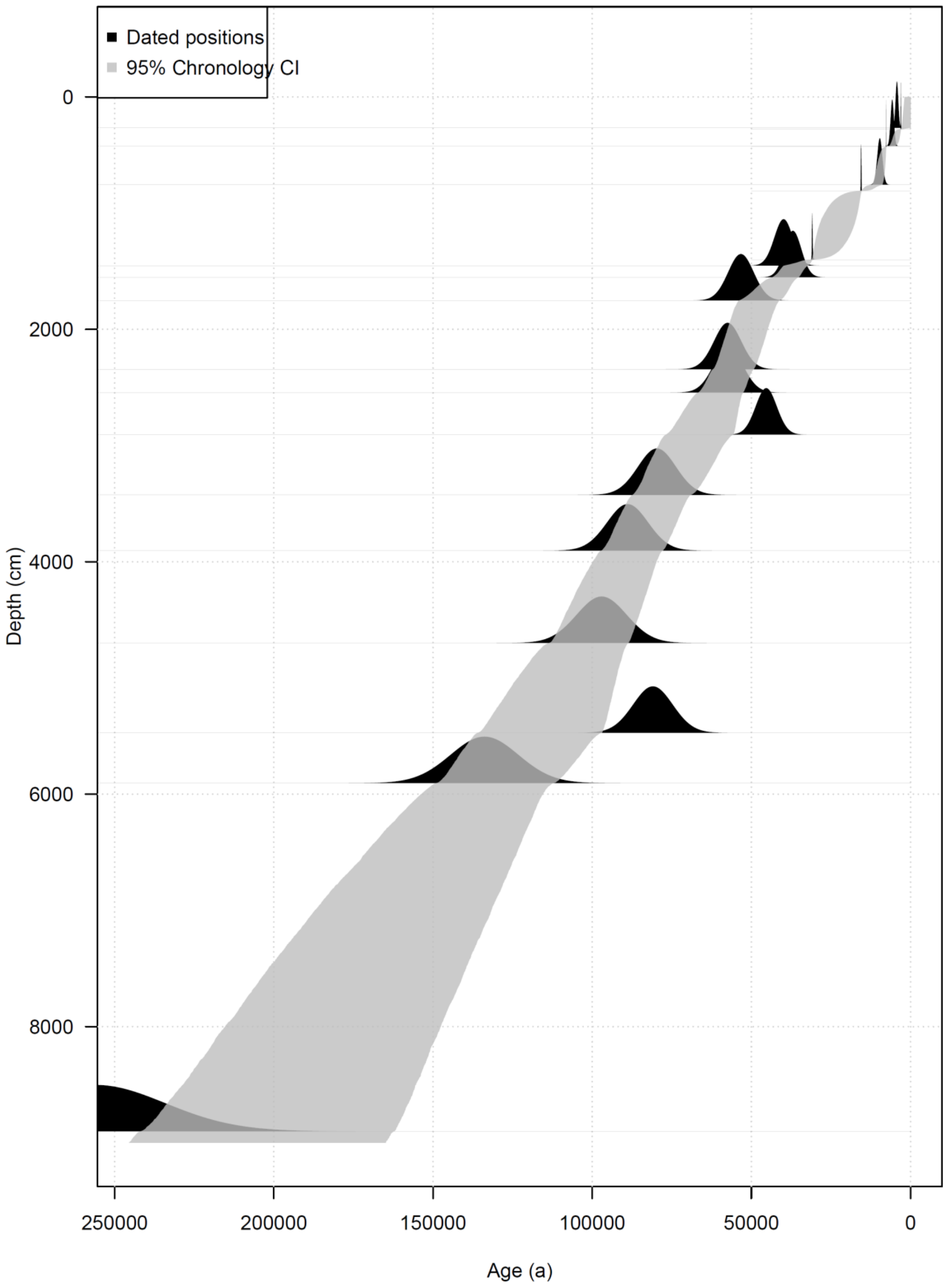

Supplementary Figure 4: Bayesian age-depth model constructed for Lake Tana using R-software package Bchron v 4.1.1[14]. The model combines 15 [post-IR] $IRSL_{225}$ ages (Supplementary Table 2) entered as normally distributed ages, and four bulk sediment radiocarbon ages (SUERC-20086, SUERC-20087, SUERC-20090, SUERC-16457; Supplementary Table 1) calibrated using Intcal13[15]. The 95% highest posterior density regions (grey shading) indicate the uncertainty of the ages assigned to the samples between the dated depths, the positions of which are shown by horizontal lines.

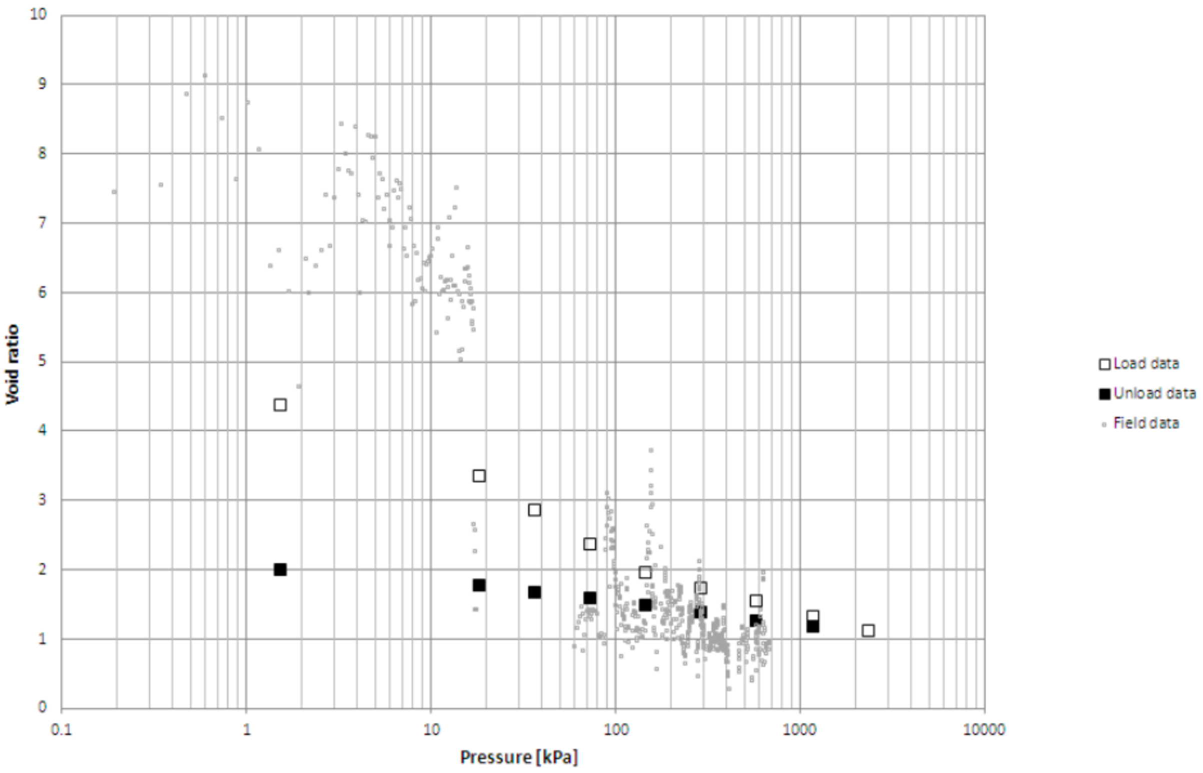

Supplementary Figure 5: Load and unload void ratio – log pressure (stress) data from the instrumented oedometer test and derived from the moisture content data of the core. An explanation of the conversion of moisture content – depth to void ratio – pressure data is given in the Supplementary Methods.

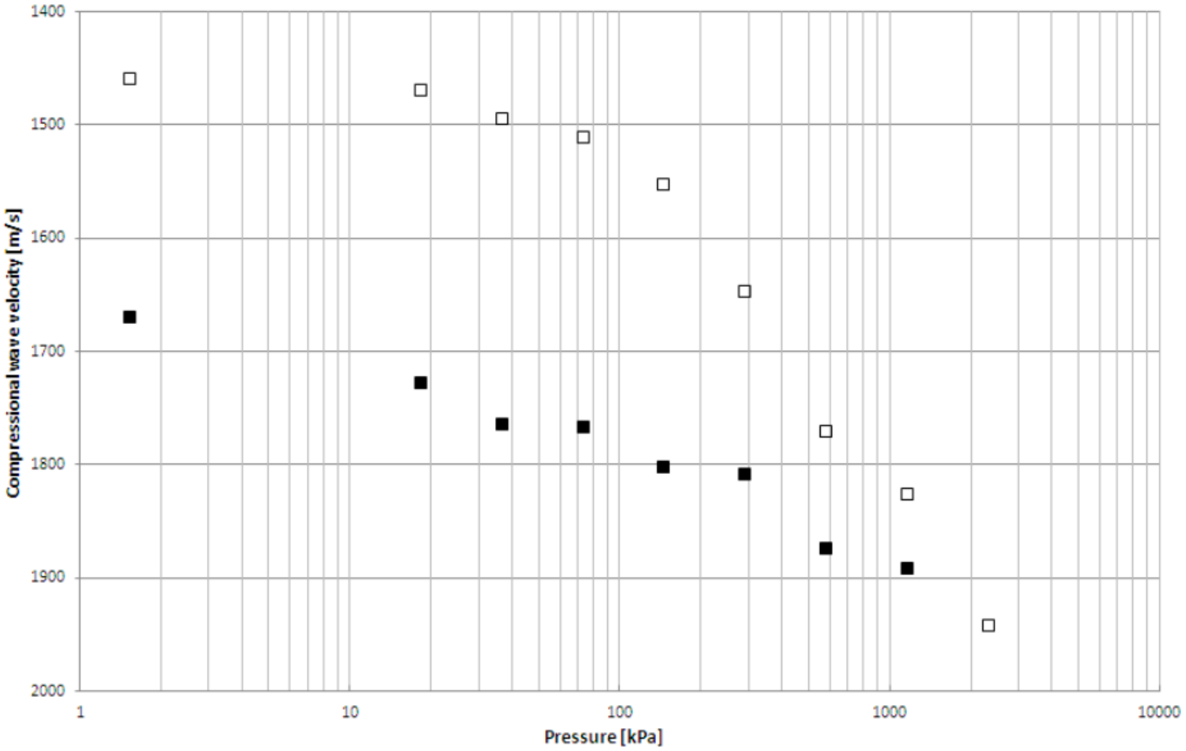

Supplementary Figure 6: Compressional wave velocity – log pressure (stress) data from the instrumented oedometer test. Open squares: load cycle data; closed squares: unload cycle data. Note the reversed scale for the velocity data.

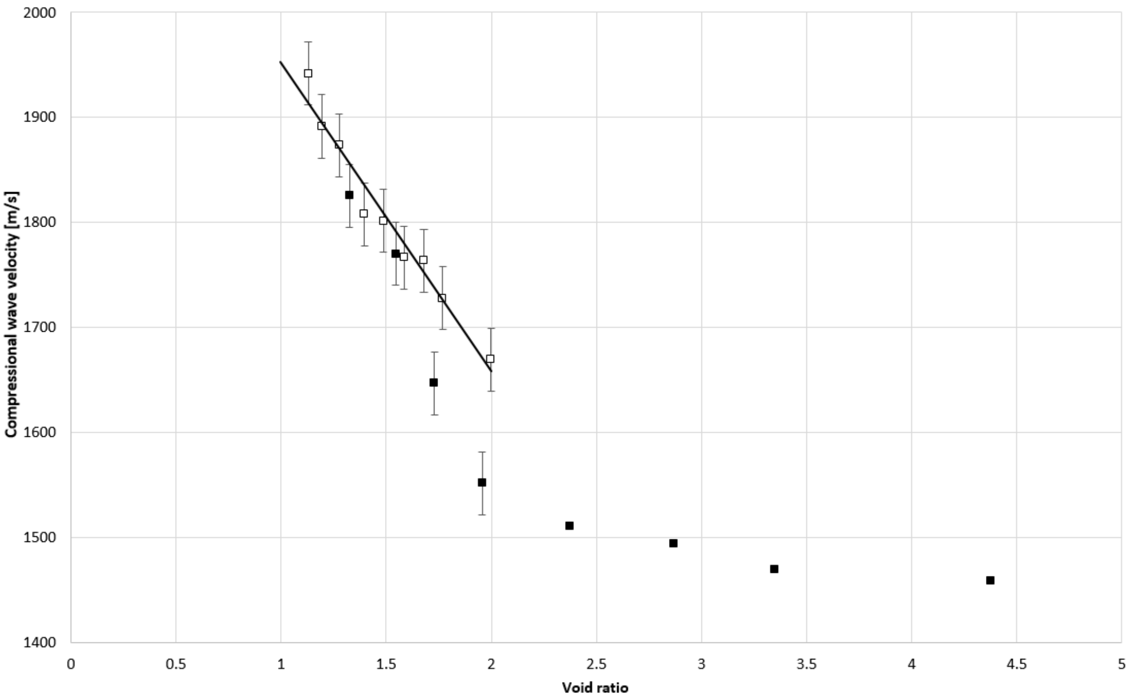

Supplementary Figure 7: A re-organisation of the instrumented oedometer data shown in Supplementary Figures 5 and 6, plotting compressional wave velocity against void ratio. Note the unload data (open squares) fll close to the load data (shaded squares), suggesting that wave velocity can be predicted from void ratio, irrespective of the stress history of the sample. Vertical bars on data points between 1 < e < 2 indicate ± 30 m/s, the range within which the sensitivity test on seismic to age conversion is made (see Supplementary Figure 8).

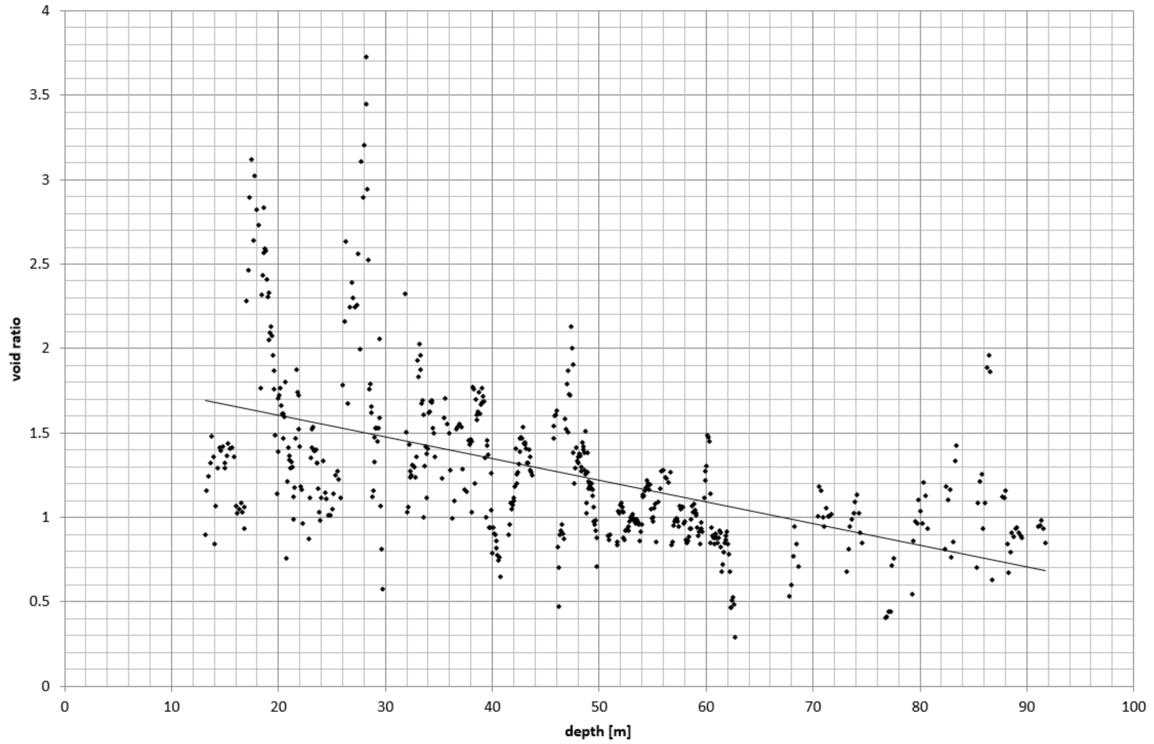

Supplementary Figure 8: Best-fit straight-line used to approximate the void ratio – depth relationship (e = 1.862 - 0.0128 z).

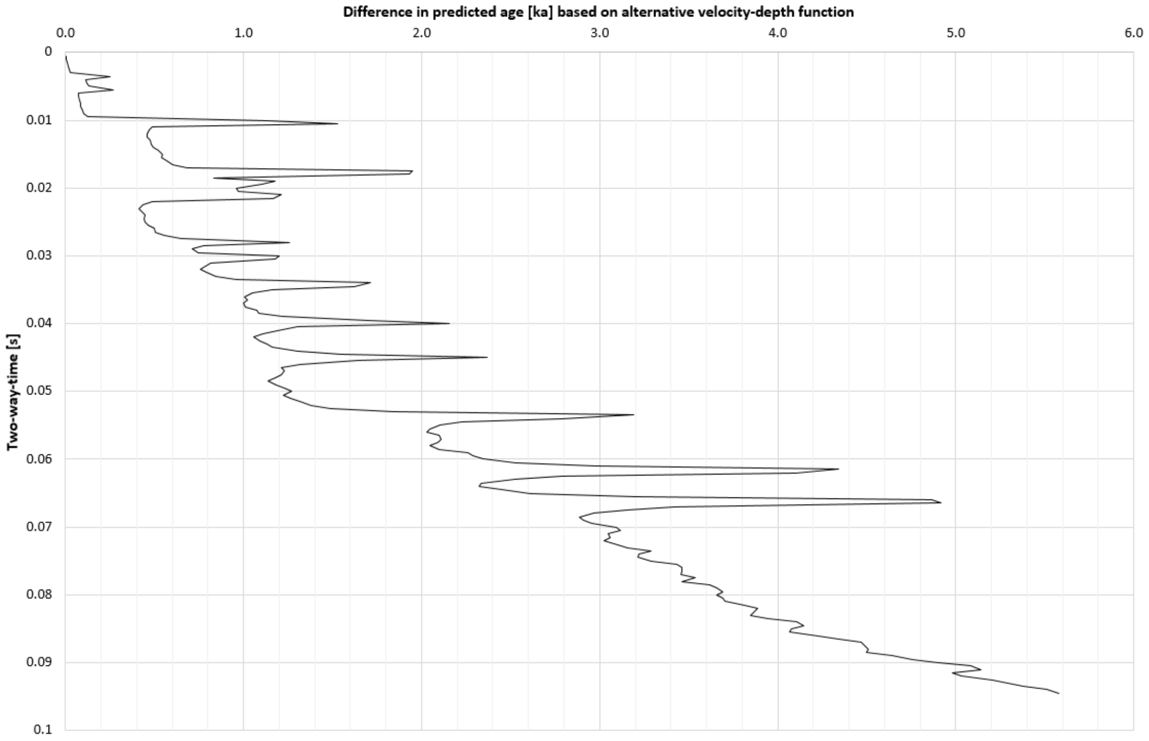

Supplementary Figure 9: Plot showing the effect of a velocity difference of ± 30 m/s on the predicted age for the range of travel times encountered in this study.

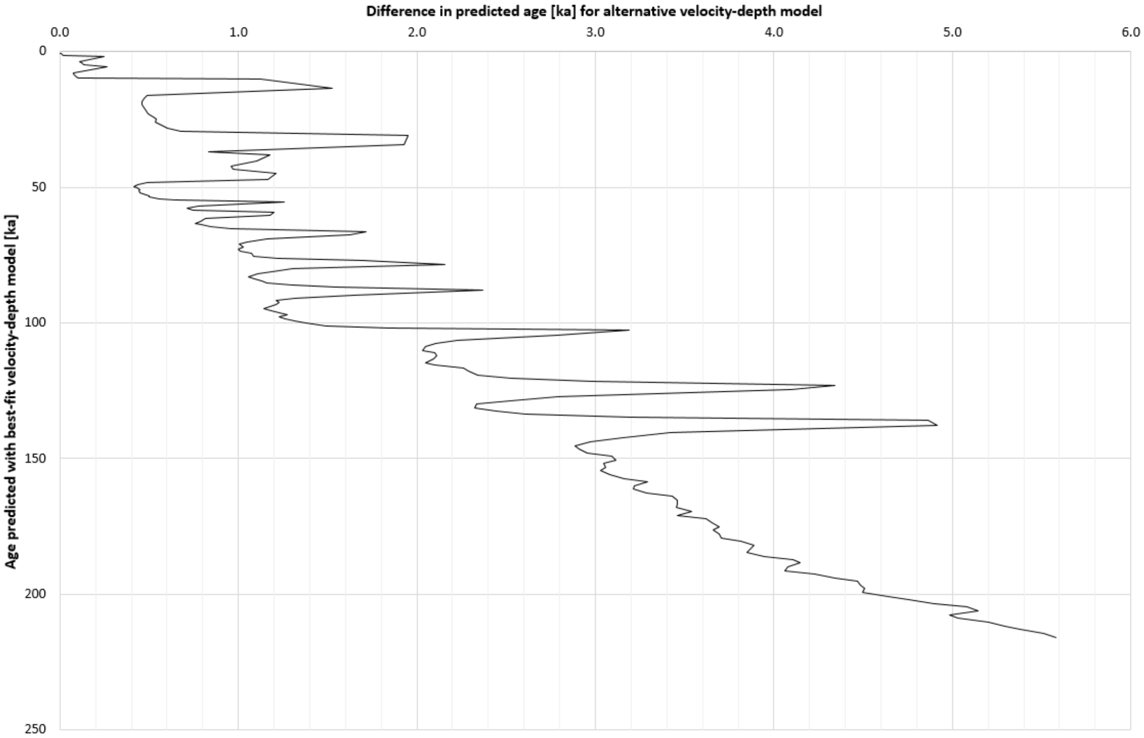

Supplementary Figure 10: Similar to Supplementary Figure 9, except that the y-axis is changed to age on the basis of the originally assumed velocity-depth model.

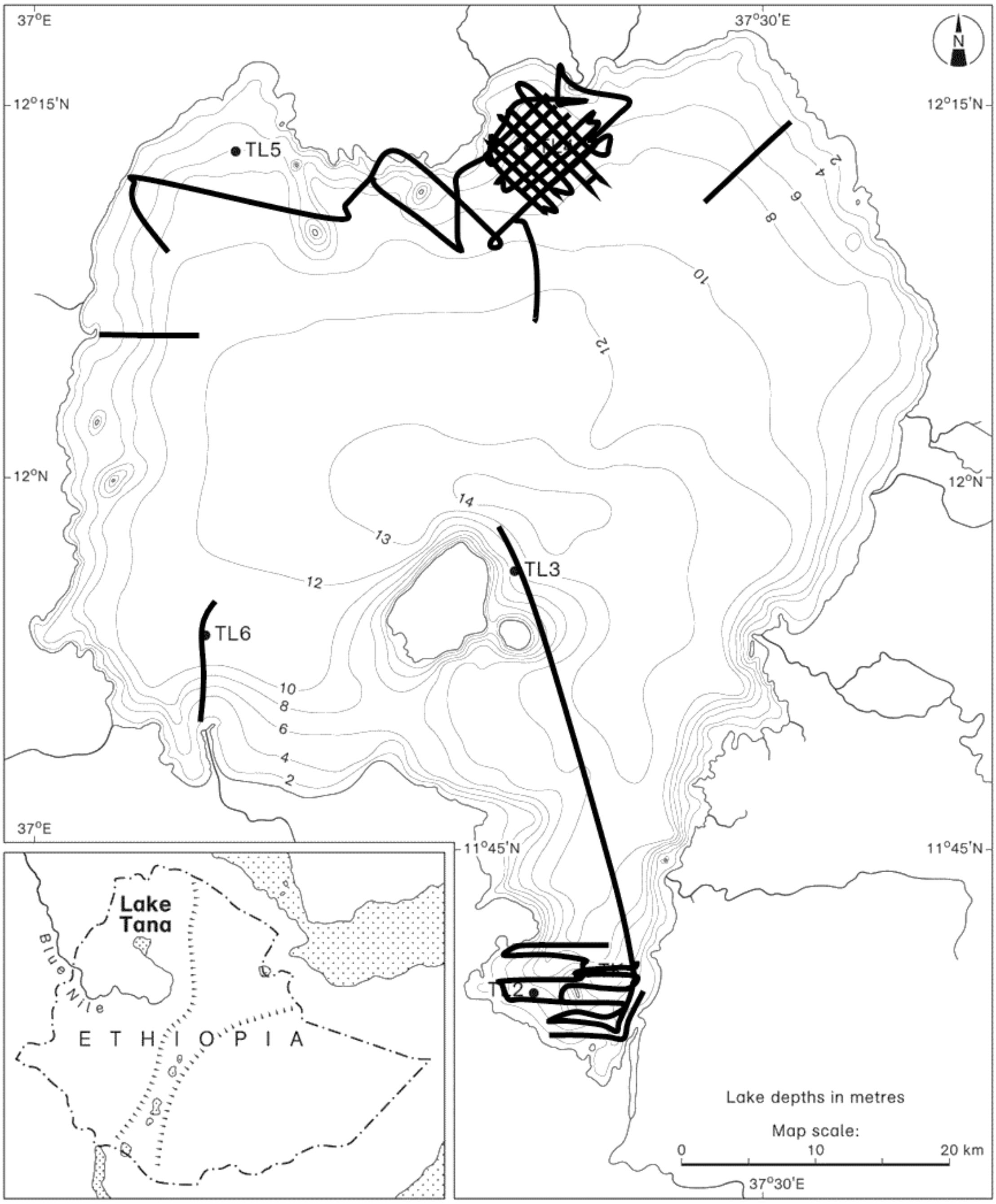

Supplementary Figure 11: Lake Tana: location of seismic acquisition lines; the core PT07 drill site is located at the centre of the grid. Survey lines to the south were acquired previously. Map generated in Adobe Illustrator CS6 Version:16.0.4. https://www.adobe.com/uk/

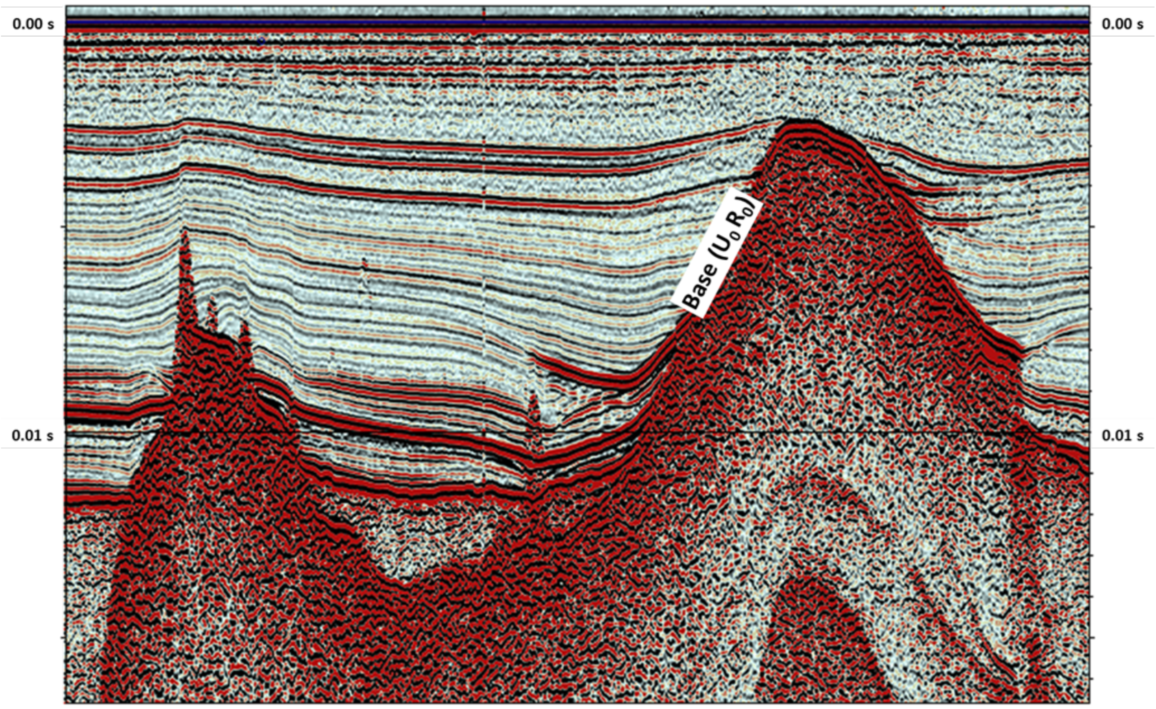

Supplementary Figure 12: Typical seismic section from a near-coast profile showing seismic basement (U_0R_0) as a high amplitude reflector, onto which subsequent strata onlap. Two-way-travel time (s) shown on vertical axes. Image represents c. 700 m distance, 14 m depth range. Seismic data of Figs 12- 16 acquired using SonatWiz, v5, Chesapeake Inc. (https://chesapeaketech.com/) and processed with Kingdom, IHS Markit https://kingdom.ihs.com/

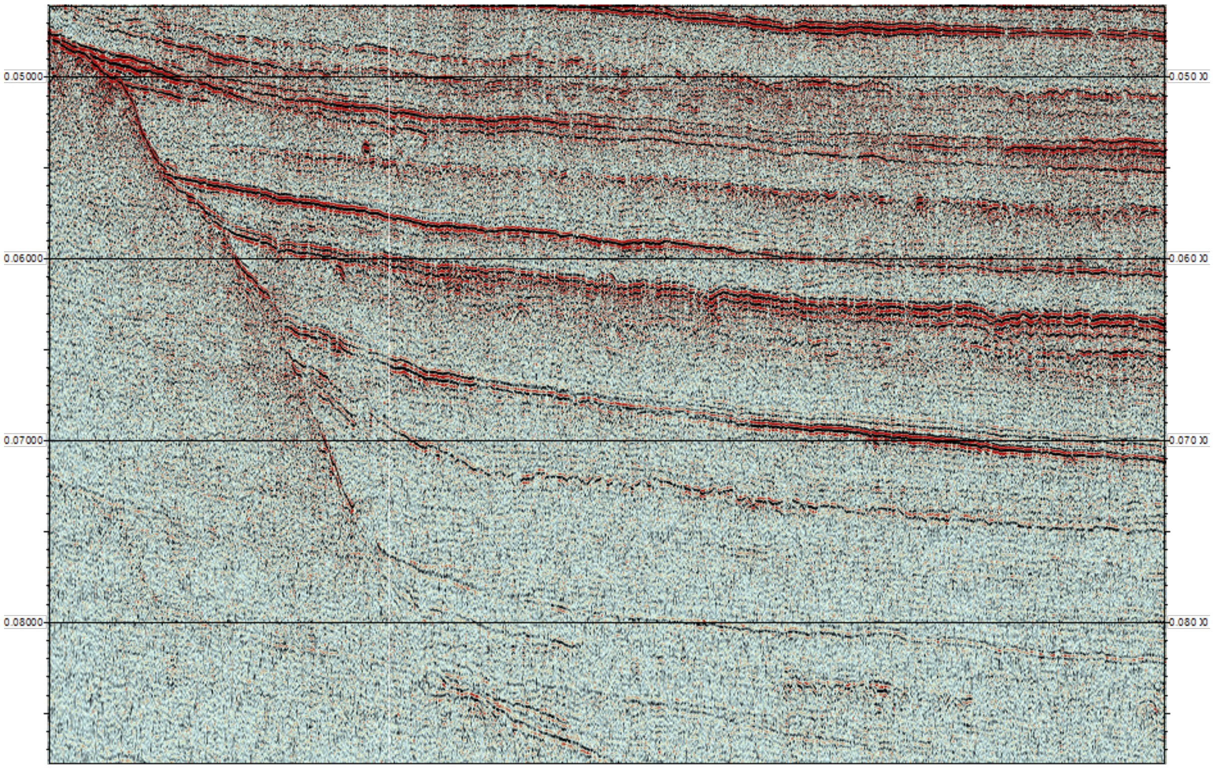

Supplementary Figure 13: Seismic Unit 1, the basin fill facies – showing broadly conformable reflectors, dipping at a shallow angle in a lakeward direction. Two-way-travel time (s) shown on vertical axes. Image represents c. 1100 m distance, 40 m depth range.

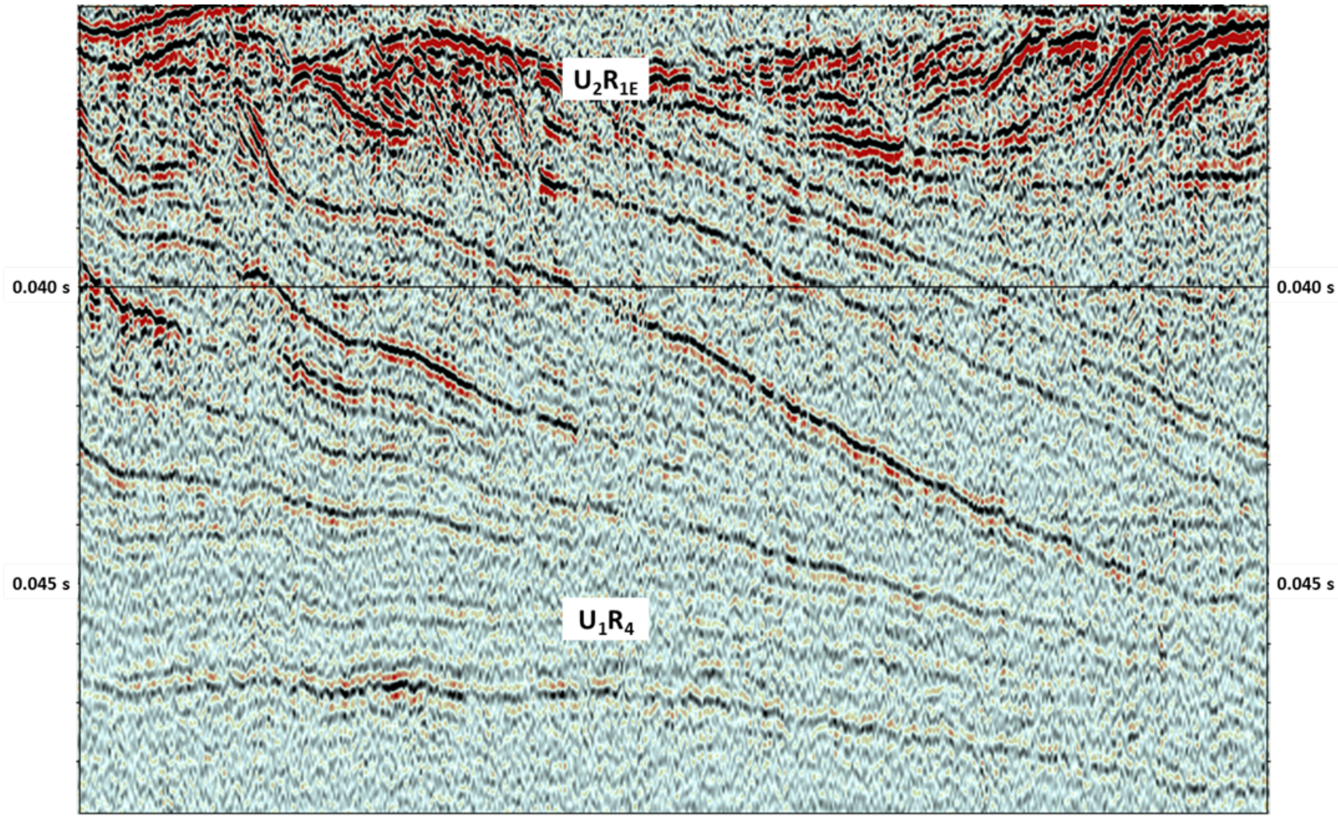

Supplementary Figure 14: Detail of the lower Sequence sub-Unit 2a, sigmoidal and oblique parallel clinoform facies. Upper termination either erosional truncation or toplap up to reflector U_2R_{1E} (P4 in [14]). Lower termination inferred by extrapolation to be downlap towards basal reflector U_1R_4. Two-way-travel time (s) shown on vertical axes. Image represents c. 700 m distance, 14 m depth range. Details of seismic reflectors and seismic units are given in Supplementary Table 3.

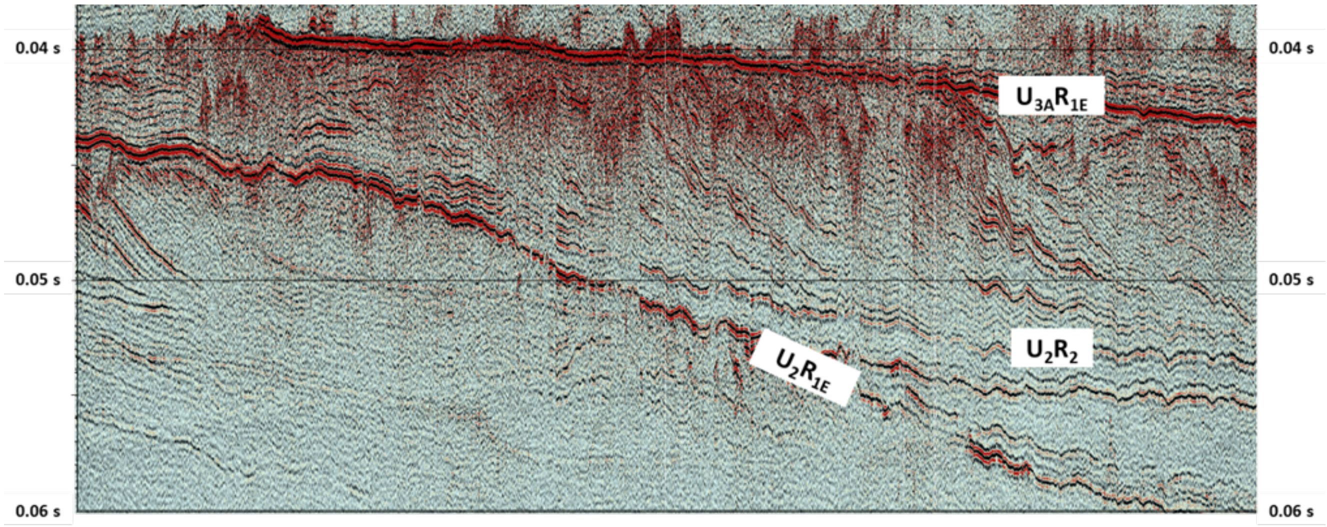

Supplementary Figure 15: Detail of the upper Sequence sub-unit 2b showing a downward, offshore shift in onlap onto basal reflector U_2R_2. Two-way-travel time (s) shown on left-hand margin. Image represents c. 4500 m distance, 20 m depth range. Details of seismic reflectors and seismic units are given in Supplementary Table 3.

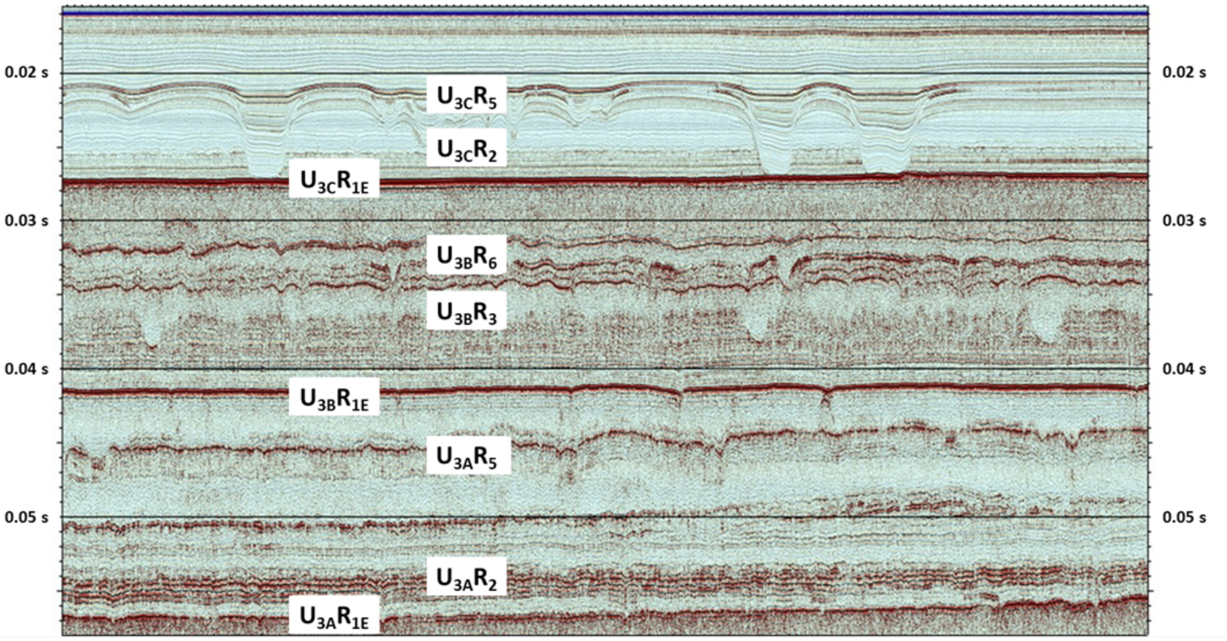

Supplementary Figure 16: Unit 3, with sub-units 3a, 3b and 3c, and high amplitude, continuous basal reflectors $U_{3A}R_{1E}$ (P3), $U_{3B}R_{1E}$ (P2) and $U_{3C}R_{1E}$ (P1). Important internal reflectors are identified as: $U_{3A}R_2$, incipient lake fill; $U_{3A}R_5$ downcutting channel with diffuse erosion surface; $U_{3B}R_3$ downcutting channel with diffuse erosion surface; $U_{3B}R_6$ top of triplet of chaotic events. Two-way-travel time (s) shown on vertical axes. Image represents c. 850 m distance, 32 m depth range. Details of seismic reflectors and seismic units are given in Supplementary Table 3.

a **b**

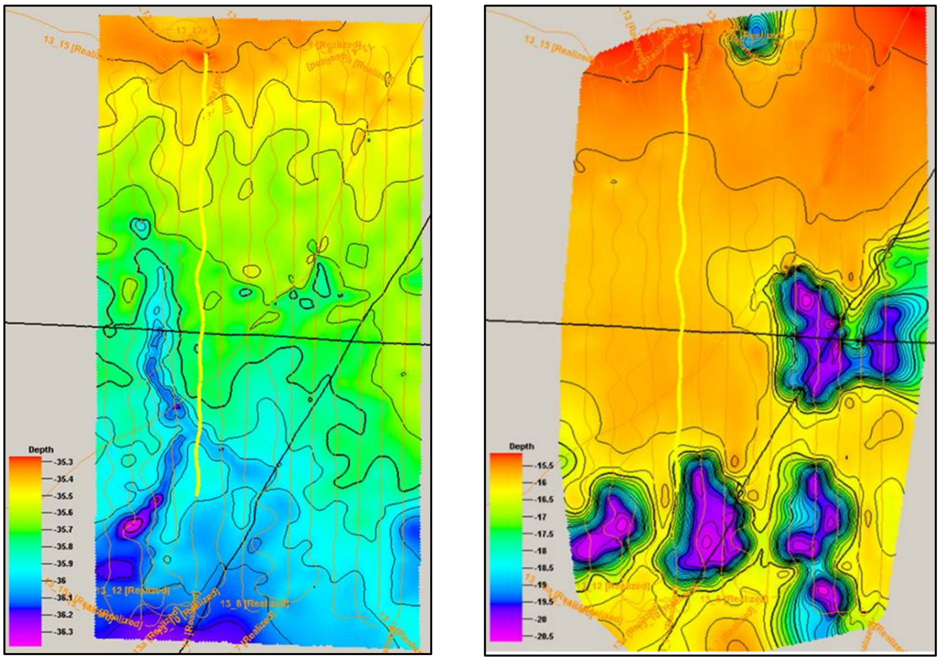

Supplementary Figure 17: (**a**) The cut-and-fill facies mapped across the densely sampled core site showing clear coherent channelling in sub-unit 3a. (**b**) Sub-unit 3c showing cut-and-fill facies as isolated basins rather than a channel complex. Depth contours in metres from lake surface.

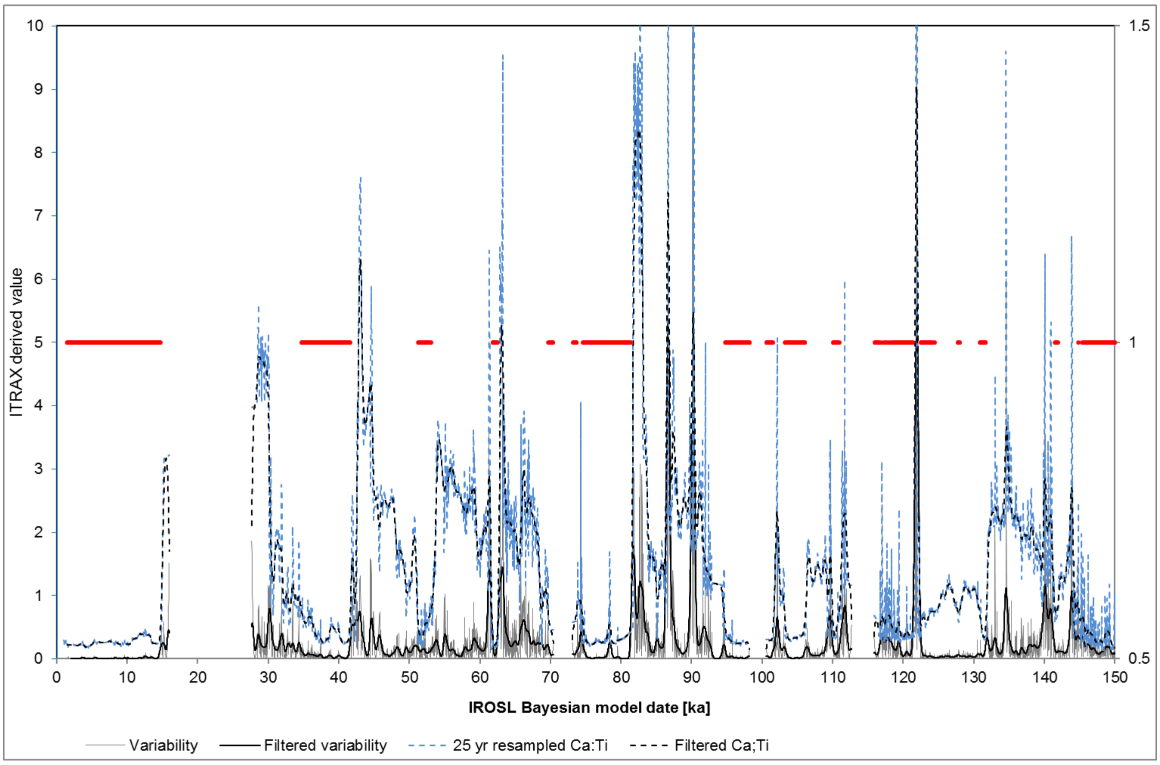

Supplementary Figure 18: Stable moist intervals (red bars) defined in the Ca/Ti record of core PT07, by methods adapted from[23].

Supplementary Table 1: Results of the radiocarbon analyses of bulk sediments from Lake Tana cores 07TL1 and PT07-2.

Sample identifier	Stratigraphic position (cm)	$\delta^{13}C_{VPDB}$ ‰	Laboratory code	% modern ^{14}C	+/- 1σ % modern ^{14}C	^{14}C yrs BP	+/- 1σ ^{14}C yrs BP
07TL1 / 3 / 279cm	279.0	-18.1	SUERC-20086	69.72	0.31	2898	35
07TL1 / 5 / 428cm	428.0	-16.1	SUERC-20087	42.78	0.20	6821	38
07TL1 / 9 / 809cm	809.0	-16.5	SUERC-20090	19.87	0.12	12980	47
PT07-2 / 4 / 1400 cm	1400.0	-20.0	SUERC-16457	3.63	0.09	26634	208

Supplementary Table 2*:* Lake Tana: luminescence sample details, equivalent dose, dose rate, and [post-IR] IRSL ages for polymineral 4-11 µm diameter grain

Core Number	ALRL[a] Sample No.	*Depth down-core (m)*	*No. aliquots used for D_e*	***Equivalent Dose, D_e (Gy)***[b]	*Water content*[c]	*Bulk K (%)*[d]	*Bulk U (ppm)*[d]	*Bulk Th (ppm)*[d]	*Cosmic (Gy/ka)*[e]	***Total dose rate (Gy/ka)***[f]	***pIRIR$_{225}$ Age (ka)***[g]
07TL1-3	*134/1*	2.86 ± 0.03	24	**2.92 ± 0.19**	280 ± 28	0.80 ± 0.04	1.62 ± 0.08	7.08 ± 0.35	0.084 ± 0.008	**0.68 ± 0.05**	**4.28 ± 0.43**
07TL1-5	*134/2*	4.22 ± 0.03	22	**5.15 ± 0.20**	240 ± 24	0.79 ± 0.04	2.21 ± 0.11	9.31 ± 0.47	0.076 ± 0.008	**0.90 ± 0.07**	**5.75 ± 0.51**
07TL1-8	*134/3*	7.55 ± 0.03	22	**7.65 ± 0.25**	240 ± 24	0.81 ± 0.04	1.79 ± 0.09	8.21 ± 0.41	0.060 ± 0.006	**0.79 ± 0.06**	**9.66 ± 0.81**
PT07-2/5a	*134/4*	14.52 ± 0.03	22	**69.05 ± 1.77**	50 ± 5	0.96 ± 0.05	1.49 ± 0.07	7.83 ± 0.39	0.040 ± 0.004	**1.73 ± 0.12**	**39.9 ± 2.9**
PT07-2/5b	*134/5*	15.52 ± 0.03	12	**66.29 ± 1.70**	50 ± 5	1.02 ± 0.05	1.42 ± 0.07	8.40 ± 0.42	0.038 ± 0.004	**1.79 ± 0.12**	**37.0 ± 2.7**
PT07-2/6a	*134/6*	17.52 ± 0.03	22	**83.76 ± 2.26**	50 ± 5	0.81 ± 0.04	1.61 ± 0.08	6.68 ± 0.33	0.034 ± 0.003	**1.57 ± 0.11**	**53.3 ± 4.1**
PT07-2/8a	*134/7*	23.44 ± 0.03	22	**101.14 ± 2.60**	50 ± 5	0.96 ± 0.05	2.10 ± 0.11	6.14 ± 0.31	0.026 ± 0.003	**1.76 ± 0.12**	**57.5 ± 4.3**
PT07-2/8c	*134/8*	25.44 ± 0.03	17	**85.07 ± 2.02**	45 ± 5	0.65 ± 0.03	1.46 ± 0.07	6.87 ± 0.34	0.023 ± 0.002	**1.49 ± 0.11**	**57.3 ± 4.5**
PT07-2/9e	*134/9*	29.06 ± 0.03	22	**76.04 ± 1.56**	55 ± 6	0.98 ± 0.05	1.95 ± 0.10	6.35 ± 0.32	0.020 ± 0.002	**1.68 ± 0.12**	**45.3 ± 3.3**
PT07-2/11c	*134/10*	34.25 ± 0.03	22	**117.11 ± 2.40**	60 ± 6	0.81 ± 0.04	1.26 ± 0.06	8.25 ± 0.41	0.000 ± 0.000	**1.46 ± 0.11**	**79.7 ± 6.0**
PT07-2/13b	*134/11*	39.04 ± 0.03	22	**139.41 ± 3.30**	60 ± 6	0.99 ± 0.05	1.40 ± 0.07	7.67 ± 0.38	0.000 ± 0.000	**1.56 ± 0.11**	**88.9 ± 6.5**
PT07-2/16a	*134/12*	46.99 ± 0.03	22	**151.79 ± 3.75**	60 ± 6	0.75 ± 0.04	1.74 ± 0.09	7.81 ± 0.39	0.000 ± 0.000	**1.55 ± 0.12**	**97.0 ± 7.9**
PT07-2/19a	*134/13*	54.72 ± 0.03	22	**152.28 ± 4.42**	40 ± 4	0.92 ± 0.05	1.84 ± 0.09	7.37 ± 0.37	0.000 ± 0.000	**1.88 ± 0.14**	**80.9 ± 6.3**
PT07-2/20b	*134/14*	59.05 ± 0.03	15	**260.72 ± 9.63**	35 ± 4	0.94 ± 0.05	1.68 ± 0.08	7.66 ± 0.38	0.000 ± 0.000	**1.95 ± 0.14**	**134 ± 11**
PT07-2/31b	*134/15*	89.02 ± 0.03	15	**438.91 ± 10.73**	35 ± 4	0.54 ± 0.03	1.87 ± 0.09	7.57 ± 0.38	0.000 ± 0.000	**1.71 ± 0.14**	**256 ± 22**

[a] Aberystwyth Luminescence Research Laboratory sample number. [b] The D_e is calculated using the weighted mean, and the error calculated is the standard error on the mean for all samples. [c] Water content is expressed as the % of dry mass of sediment, and calculated as the mean of the measured water content values (determined every 8cm depth) for sediments spanning ± 30cm of the sample taken for luminescence dating.[d] Samples were ground and fused with lithium metaborate, prior to ICP-MS measurements to determine uranium (U) and thorium (Th) concentrations, and ICP-OES to determine potassium (K) concentrations. A 5% uncertainty was attributed to each determination based on the reproducibility of analyses over three batches of measurements. [e] The contribution from cosmic rays was calculated according to [24], using a water depth of 12.5 m overlying the sediment depth down-core, and assigned an uncertainty of 10%. The maximum depth for which the cosmic dose rate was calculated was 29 m; below this depth the data generated are likely to become increasingly unreliable [24]). Sensitivity analysis of the effect of changing sample depth over time (conducted using a fixed water depth of 12.5 m, and comparing the dose rate calculated for the deepest sample both at its present depth and also at the current depth of the uppermost sample in the study) showed that even the greatest change in cosmic dose rate had less than 5% effect on the overall dose rate. [f] Dose rates were calculated using the values of [25]. An *a*-value of 0.08 ± 0.02 was assumed[26]. [g] [post-IR] IRSL$_{225}$ ages are calculated prior to rounding, expressed as thousands of years before 2010 AD, and shown to three significant figures.

Supplementary Table 3: Summary table of interpreted seismic stratigraphy. Two-way travel times picked directly from seismic data; depths calculated as described above; ages ascribed from BChron age-depth model. Depths and ages refer to unit tops. Double undulating line denotes major, unambiguous, erosion surface; single line denotes minor erosion surface leading to cut-and-fill facies.

**Reflector**<br>**Full Name (name given in [21])**	**Two Way Time [ms]**	**Depth [m]**	**Age [ka]**	**Facies**	**Unit**	**Interpretation**
$U_{3C}R$	3.66	2.75	**2.7**	High amplitude horizontal event	Unit 3c	Change in consolidation state or lithology. Change in depositional rate / environment.
$U_{3C}R_5$	5.17	3.88	**5.1**	Uppermost event of cut-and-fill facies		Resumption of lake-wide deposition
				Cut and fill facies forming isolated ponds.		Lowered lake level
$U_{3C}R_4$	6.59	4.94	**8.2**	Uppermost portion of transparent facies		
				Generally seismically transparent. Occasional low amplitude horizontal events.		Lake infill; little change in environment of deposition.
$U_{3C}R_3$	8.65	6.49	**9.3**	Hummocky low amplitude reflector		
$U_{3C}R_2$	9.95	7.46	**10.1**	High amplitude reflector. Top of horizontal banded reflectors. Strong down-cutting		End of incipient post-erosion infill
$U_{3C}R_{1\,E}$ (P1)	10.99	8.26	**16.0**	High amplitude reflector. **Erosion surface**		Sub-aerial exposure.
				Chaotic, high amplitude return	Unit 3b	Coarse grained material?
$U_{3B}R_6$	15.01	11.77	**25.0**	Upper of triplet		Change in consolidation state or lithology. Change in depositional rate / environment.
$U_{3B}R_5$	16.50	13.07	**28.3**	Middle of triplet		
$U_{3B}R_4$	17.88	14.28	**33.3**	Sub-parallel, spaced reflector set with low amplitude return between events. Lower of triplet		
				Seismic transparent horizon		Uniform, fine grained deposition and consolidation.
$U_{3B}R_3$	20.50	16.58	**43.5**	Medium amplitude, chaotic sub-unit. Channel- like diffuse, **erosional upper surface**		Likely sub-aerial exposure.
$U_{3B}R_2$	21.99	17.90	**48.3**	Parallel, horizontal, high amplitude reflector sets		Incipient lake fill

$U_{3B}R_{1\,E}$ (P2)	25.20	20.74	**52.0**	High amplitude reflector. **Erosion surface**		Sub-aerial exposure.
				Seismic transparent horizon	Unit 3a	Rapid fill?
$U_{3A}R_5$	27.77	23.03	**55.1**	Sub-parallel, slightly hummocky spaced reflector with low amplitude return between events		Weak, “channel-like” diffuse erosional surface
$U_{3A}R_4$	32.08	26.90	**63.3**	Sub-parallel, slightly hummocky spaced reflector with low amplitude return between events		Weak, “channel-like” diffuse erosional surface
$U_{3A}R_3$	33.52	28.19	**65.4**	Diffuse, undulating, low-medium amplitude reflectors above low-medium amplitude, chaotic zone		
$U_{3A}R_2$	36.53	30.92	**72.0**	Parallel, horizontal, high amplitude reflector sets. Medium-high amplitude upper reflector		Incipient lake fill?
$U_{3A}R_{1\,E}$(P3)	38.93	33.11	**76.0**	High amplitude reflector. **Erosion surface**		Sub-aerial exposure.
				Parallel to sub-parallel, dip to lake centre. Stratigraphy becomes more complex in nature at shallower depth.	Unit 2b	**Prograding wedge formation**
U_2R_2	45.68	39.30	**90.0**			Base of progradation Phase II
				Parallel to sub-parallel, horizontal reflectors, initiated after lakeward and down shift in onlap.		Re-establishment of deposition after the underlying erosion event.
$U_2R_{1\,E}$ (P4)	52.66	45.80	**101.2**	High amplitude reflector. **Erosion surface**		Sub-aerial exposure.
				Parallel to sub-parallel, dip to lake center, near shore discontinuous, undulating	Unit 2a	**Prograding wedge formation**
U_1R_4	60.66	53.34	**120.7**	Near-horizontal low amplitude reflector	Unit 1	Closest equivalent reflector at core site that forms downlap surface at prograding wedge area of the survey i.e. base of wedge, Phase I
U_1R_3 (P5)	62.78	55.36	**128.1**	Medium-low amplitude reflector, traceable across all data.		Change in consolidation state or lithology. Change in depositional rate / environment.
				Parallel to sub-parallel, horizontal internal reflectors		Quiescent basin fill

U_1R_2 (P6)	79.01	71.04	**173.6**	Low amplitude near horizontal reflector, traceable across all data.		Change in consolidation state or lithology. Change in depositional rate / environment.
				near horizontal sub-parallel, continuous, low to medium amplitude, onlap onto basement		Quiescent basin fill.
$U_1R_{1\ E}$	80.91	72.88	**178.9**	Hummocky reflector		Erosion surface? Sub-aerial exposure?
				near horizontal sub-parallel, continuous, low to medium amplitude, onlap onto basement		Quiescent basin fill.
U_0R_0						Seismic basement (not visible at core site)
